# Design, Synthesis, and Toxicological Activities of Novel Insect Growth Regulators as Insecticidal Agents against *Spodoptera littoralis* (Boisd.)

**DOI:** 10.1021/acsomega.2c05977

**Published:** 2022-12-20

**Authors:** Antar A. Abdelhamid, Safwat A. Aref, Nabila A. Ahmed, Ahmed M. M. Elsaghier, Fawy M. Abd El Latif, Sameera N. Al-Ghamdi, Mohamed A. Gad

**Affiliations:** † Department of Chemistry, Faculty of Science, Sohag University, Sohag 8252, Egypt; ‡ Chemistry Department, Faculty of Science, Albaha University, Albaha 1988, Saudi Arabia; § Research Institute of Plant Protection, Agricultural Research Center, Giza 12112, Egypt; ∥ Chemistry Department, Faculty of Science, Aswan University, Aswan 81528, Egypt

## Abstract

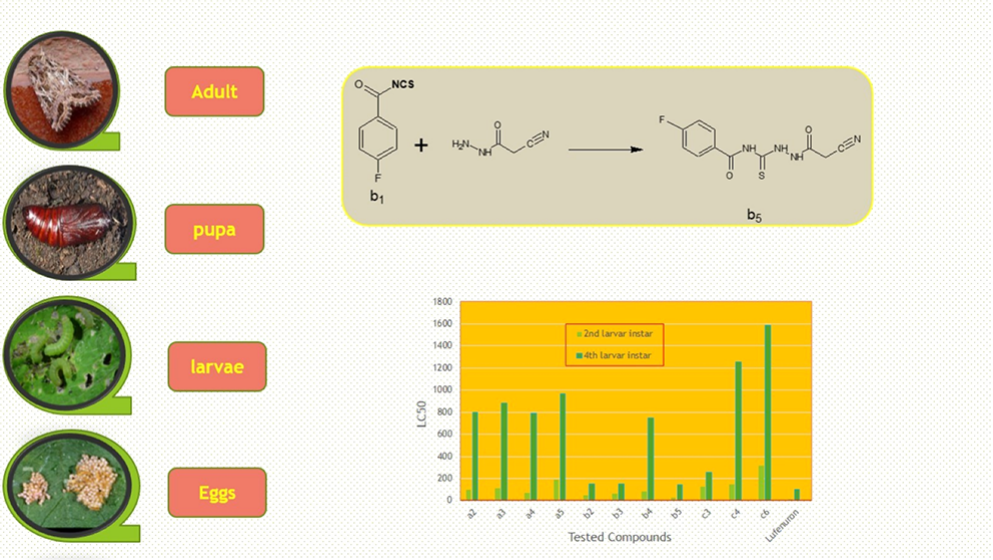

As a result of some major problems that come from using insecticides, the use of safe alternatives to these pesticides has become very necessary. Thus, a novel series of predicted toxicologically active urea, thiourea, thiosemicarbazide, oxadiazole, pyrazole, and triazine derivatives have been synthesized in a pure form to be lufenuron analogues as insect growth regulators which were screened and examined against *Spodoptera littoralis* (Boisd). The structure of synthesized compounds was established by means of spectroscopic and elemental analyses. Compounds **b5**, **b2**, **b3**, and **a4** showed high insecticidal toxicity, and their LC_50_ values for the second larvae instar were found to be 26.63, 46.35, and 60.84 ppm, respectively, whereas the LC_50_ value for lufenuron as a reference insecticide was 17.01 ppm.

## Introduction

*Spodoptera littoralis* (Boisduval, 1833) is a species of moth in the family Noctuidae;^1^ it is found widely in Africa, Mediterranean Europe, and Middle Eastern countries.^2^ It is known that the cotton leaf worm leads to great financial losses for many countries.^3^*S. littoralis* is a highly dangerous polyphosphorous moth that feeds on more than 100 species of plants of high economic value, including cotton, potatoes, corn, and vegetables.^4,5^ In recent years, urea and thioureas that are important sulfur- and nitrogen-containing compounds have proven to be important substances in drug research.^6,7^ The derivatives of urea and thioureas such as *N*-nitrosoureas, benzoylureas, benzoylthioureas, and diarylsulfonylureas have a wide range of activities against leukemias and solid tumors in which the most chapter of anticancer agents.^8^ Urea and thiourea derivatives are well-established important structures in medicinal and synthetic chemistry.^9^ The structural forms of this group constitute a common framework for a large variety of drugs and biologically and chemically active compounds which are used for their therapeutic and pharmacological properties.^10−12^ Acyl urea and acyl thiourea compounds are known for their superior activity in insecticides and plant growth-regulating activity intermediaries.^13,14^ In addition, heterogeneous nitrogen and sulfur are very important in the manufacture of active compounds such as herbicides and pesticides in the agrochemical industry.^15^ Moreover, the structure of compounds that contain an internal N–O or N–S bond can benefit plant absorption and metabolism.^16,17^ In continuation of our research theme, we wish to report the synthesis and characterization of new urea and thiourea derivatives which were assessed as insecticidal agents against *S. littoralis* instar larvae.

## Materials and Methods

All prepared target compounds were estimated MP by the Fisher–John mechanical technique.

### Instrumentation and Chemicals

For this study, chemicals and solvents were purchased from Sigma-Aldrich. The IR spectra of the prepared compounds were analyzed using the KBr method, and ^1^H NMR and ^13^C NMR spectra were recorded on the spectrometer model Bruker ADVANCE 400 MHz. A reference lufenuron insecticide was bought from Sigma-Aldrich. The insecticidal activity of the target synthesized compounds and lufenuron was tested against *S. littoralis* instar larvae.

### Bioassay Screening

The insecticidal bioactivity of all prepared urea, thiourea, thiosemicarbazide, oxazole, pyrazole, and triazine derivatives was screened by standard leaf dip bioassay methods.^18−25^ The results of the target compounds for laboratory tests were recorded, and the concentrations required to kill 50% (LC_50_) of *S. littoralis* larvae were determined. In this article, five concentrations of urea and thiourea derivatives and 0.1% Tween-80 were used as a surfactant. Discs (9 cm diameter) of castor bean leaves were dipped in the tested concentration for 10 s, allowed to dry, and then given to the second and fourth larvae, approximately the size; the larvae were placed in glass jars (5 lb), and each treatment was repeated three times (10 larvae each). The dunked control disks in water and Tween-80 were then transferred to the untreated ones. The larvae were fed on castor beans for 48 h and then transferred to the untreated one. The mortality was calculated after 72 h at 22 ± 2 °C and 60 ± 5% relative humidity for all synthesized compounds. The mortality was calculated using Abbott’s formula.^27^ The measurements’ mortality relapse line was measurably dissected by probity analysis.^28^ The harmfulness index was determined by Sun’s equations.^26^

### Breeding Larva Insects

*S. littoralis* insects were brought from fields of the agricultural research center farm at the Sohag branch during the 2020/2021 season, and the activity of the prepared compounds and lufenuron reference insecticide was tested against the *S. littoralis* insects.

### Statistical Analysis

The mortality data of larval insects were calculated by using probit analysis via a statistical (LDP-line) equation which was used to calculate the LC_50_ values with 95% fiducially limits of lower and upper confidence and slope.

## Results and Discussion

### Synthesis

Herein, target products, namely, *N*-{[2-(cyanoacetyl)hydrazinyl]carbonothioyl}furan-2-carboxamide (**a2)**, *N*-[(2-benzoyl-hydrazinyl)carbonothioyl]furan-2-carboxamide (**a3**), *N*-[5-(cyanomethyl)-1,3,4-oxadiazol-2-yl]furan-2-carboxamide (**a4**), *N*-(5-phenyl-1,3,4-oxadiazol-2-yl)furan-2-carboxamide (**a5**), 2,6-dichloro-*N*-{[2-(4-chlorobenzoyl) hydrazinyl]carbonothioyl}benzamide (**b2**), *N*-[(4-acetylphenyl)carbamothioyl]-2,6-dichlorobenzamide (**b3**), *N*-[(4-acetylphenyl)carbamothioyl]-4-fluorobenzamide (**b4**), *N*-{[2-(cyanoacetyl)hydrazinyl]carbonthioyl}-4-fluorobenzamide (**b5**), 2-(5-imino-4,5-dihydro-1*H*-pyrazol-3-yl)-*N*-phenyl hydrazinecarboxamide (**c3**), 2-(5-imino-1-phenyl-4,5-dihydro-1*H*-pyrazol3-yl)-*N*-phenylhydrazinecarboxamide (**c4**), and 1-{4-[(6-amino-1-phenyl-1,4-dihydro-1,3,5-triazin-zyl)amino]-phenyl}ethanone (**c6**), were successfully prepared; the obtained yield was 70–89% through the following steps.

### Synthesis of Arylisothiocyanate (**a1** and **b1**)

An equimolecular amount of acid chloride (2-furoyl chloride, 2,6-dichlorobenzoyl chloride, and 4-fluorobenzoyl chloride) was added dropwise with stirring to an equimolecular amount of ammonium thiocyanate in dry acetone and refluxed for 3 h to give **a1** and **b1** in a 70–89% yield.

#### Synthesis of *N*-{[2-(Cyanoacetyl)hydrazinyl]carbonothioyl}furan-2-carboxamide (**a2**), *N*-[(2-Benzoyl-hydrazinyl)carbonothioyl]furan-2-carboxamide (**a3**), *N*-[5-(Cyanomethyl)-1,3,4-oxadiazol-2-yl]furan-2-carboxamide (**a4**), and *N*-(5-Phenyl-1,3,4-oxadiazol-2-yl)furan-2-carboxamide (**a5**)

**Scheme 1:**
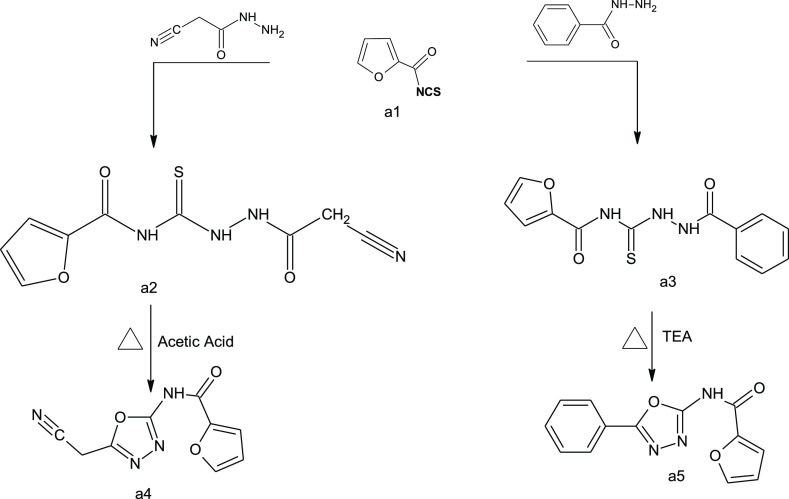
Synthesis of Oxadiazole Derivatives **a4** and **a5**

A solution of 2-furoyl isothiocyanate (freshly prepared in acetone as the solvent) was added to the amino derivative (cyanoacetohydrazide or benzhydrazide) in 15 mL of acetone; the reaction solution was refluxed for 3 h to produce compounds **a2** and **a3**, respectively, which were cyclized by refluxing in acetic acid to give **a4** and in triethylamine to give **a5** (Scheme 1).

#### Synthesis of 2,6-Dichloro-*N*-{[2-(4-chlorobenzoyl)hydrazinyl]carbonothioyl} Benzamide (**b2**), *N*-[(4-Acetylphenyl)carbamothioyl]-2,6-dichlorobenzamide (**b3**), *N*-[(4-Acetylphenyl)carbamothioyl]-4-fluorobenzamide (**b4**), and *N*-{[2-(Cyanoacetyl) hydrazinyl]carbonthioyl}-4-fluorobenzamide (**b5**)

**Scheme 2:**
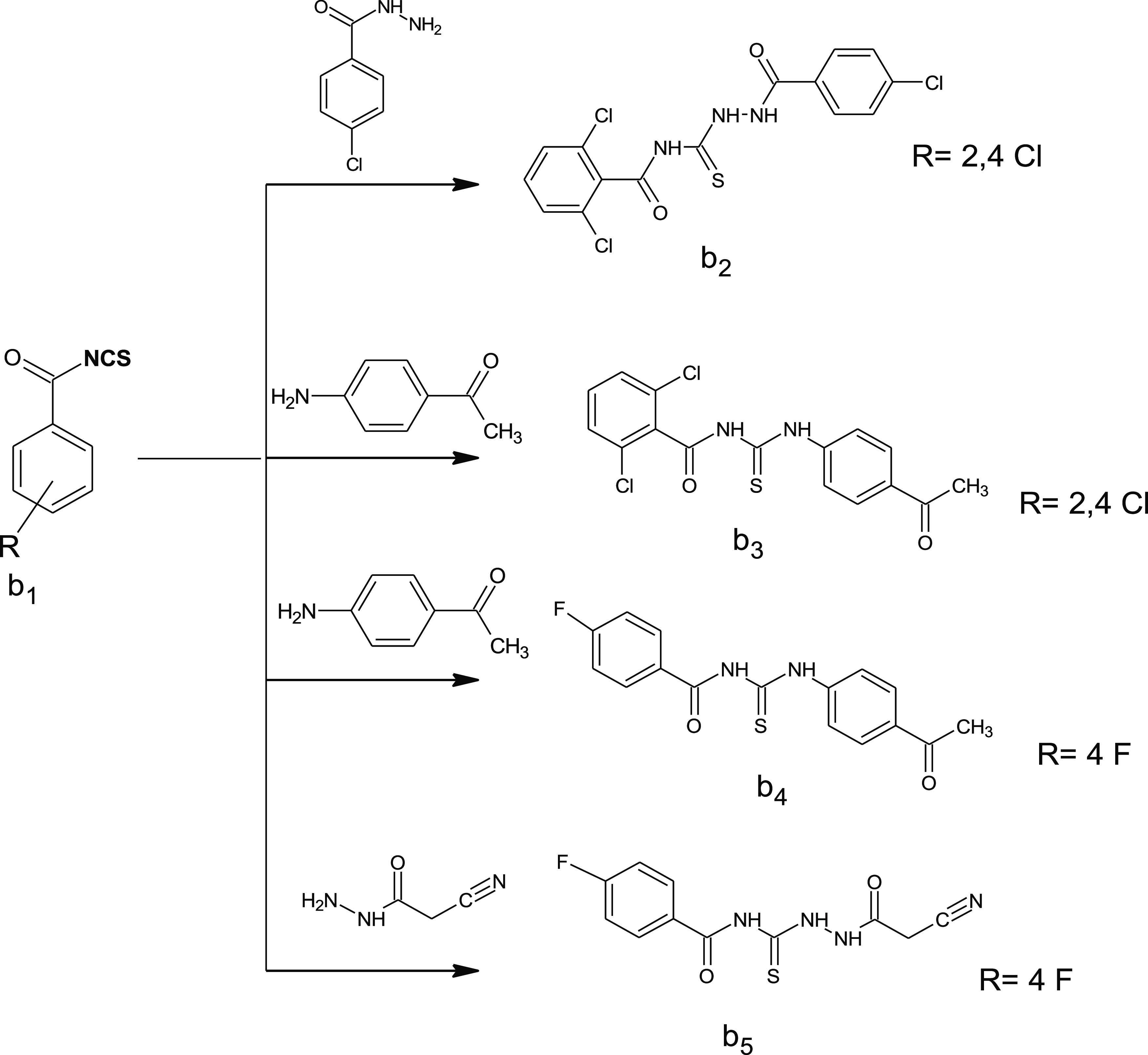
Synthesis of Thiosemicarbazide Derivatives

A solution of isothiocyanate derivatives named 2,6-dichlorobenzoylisothiocyanate or 4-fluorobenzoylisothiocyanate (freshly prepared in acetone as the solvent) was added to the amino derivative (4-chlorobenzohydrazide, 1-(4-aminophenyl)ethanone, and cyanoacetohydrazide) in 15 mL of acetone, and the reaction solution was refluxed for 3 h to give **b2**, **b3**, **b4**, and **b5** (Scheme 2).

#### Synthesis of 2-(5-Imino-4,5-dihydro-1*H*-pyrazol-3-yl)-*N*-phenyl Hydrazine-carbox-amide (**c3**), 2-(5-Imino-1-phenyl-4,5-dihydro-1*H*-pyrazol-3-yl)-*N*-phenyl-hydrazine Carboxamide (**c4**), and 1-{4-[(6-Amino-1-phenyl-1,4-dihydro-1,3,5-tri-azinyl)amino]phenyl}ethanone (**c6**)

**Scheme 3:**
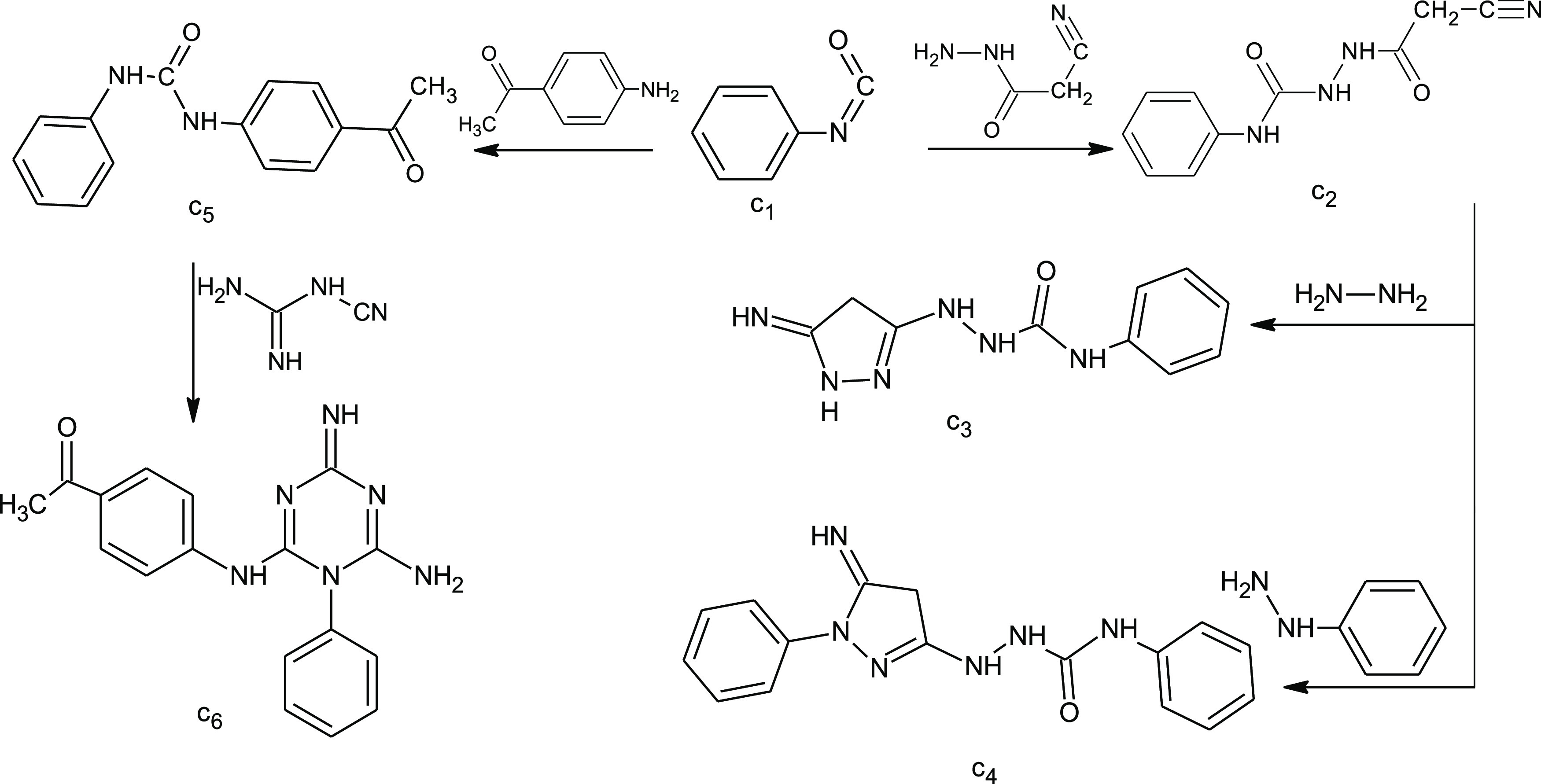
Synthesis of Pyrazole and *s*-Triazine Derivatives

The reaction of phenylisocyanate with cyanoacetohydrazide in acetone afforded **c2**, which was then reacted with hydrazine hydrate and phenyl hydrazine to give **c3** and **c4**, respectively. However, the reaction of phenylisocyanate with 4-aminoacetophenone gave **c5**, which was subsequently reacted with cyanoguanidine in a sodium ethoxide solution to give **c6** (Scheme 3).

**Scheme 4:**
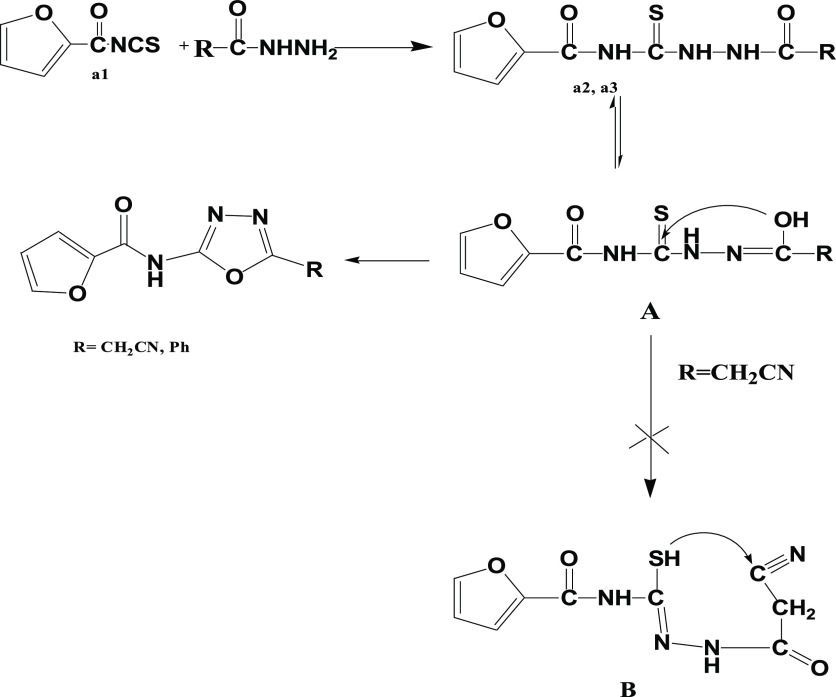
Proposed Mechanism of Formation of **a4** and **a5**

A plausible mechanism may be suggested for the formation of the target compounds **a4** and **a5** as presented in the following scheme. Thus, a nucleophilic attack of the amine function of the hydrazide to the multiple carbon atoms of the isothiocyanate group afforded the carboxamide derivatives **a2** and **a3**. Then, intramolecular nucleophilic cyclization of compounds **a2** and **a3** in their hydroxyl form A resulted in the formation of 1,3,4-oxadiazole via the elimination of H_2_S during reflux; on the other hand, in case of R=CH_2_CN, the nucleophilic attack of the SH group on the CN group did not take place; the analysis of the IR group confirmed the presence of the cyano group; also, ^1^H NMR and ^13^C NMR confirmed the presence of a free CH_2_ group (Scheme 4).

## Experimental Section

### General Procedure for the Synthesis of Compounds **a2–5**

A mixture of an equimolecular amount of ammonium thiocyanate (50 mmol) in dry acetone and 2-furoyl chloride (50 mmol) was stirred, refluxed for 3 h (2-furoyl isothiocyanate was freshly prepared in acetone as the solvent), and then added to an amino derivative (cyanoacetohydrazide or benzhydrazide) (30 mmol) in 15 mL of acetone; the reaction mixture was then refluxed for 3 h. The precipitate was collected and washed thoroughly with H_2_O and crystallized from a methanol/dichloromethane mixture (1:1).^29^

### *N*-{[2-(Cyanoacetyl)hydrazinyl]carbonothioyl}furan-2-carboxamide (**a2**)

Yellow solid (88% yield); mp 184–186 °C; IR (ν^–^, cm^–1^): 3216.09 (NH), 3107 (CH_arom_), 2922 (CH_aliph_), 2200 (CN), 1664.52 (C=O), 1580 (C=C) (Figure S1). ^1^H NMR (DMSO-*d*_6_), (δ ppm): 12.26 (s, 1H, NH_exch_), 11.50 (s, 1H, NH_exch_), 11.15 (s, 1H, NH_exch_), 8.06 (s, 1H, CH_arom_), 7.83 (s, 1H, CH_arom_), 6.75 (s, 1H, H_arom_), 3.85 (s, 2H, CH_2aliph_) (Figure S2). ^13^C NMR: 178.53, 160.39, 157.64, 148.75, 145.11, 118.22, 115.52, 113.07, 24.18. Anal. for C_9_H_8_N_4_O_3_S (252.249) (Figure S3). Calcd./found C:42.85/42.83, H: 3.20/3.18, and N: 22.21/22.20%.

### *N*-[(2-Benzoylhydrazinyl)carbonothioyl]furan-2-carboxamide (**a3**)

Brown crystals (85% yield), mp 159–161 °C; IR (ν^–^, cm^–1^): 3206.49, 3097(CH_arom_), 1667.23 (C=O), 1565.44 (C=C).^1^H NMR (DMSO-*d*_6_), (δ ppm): 9.50 (s, 1H, NH_exch_), 8.50 (s, 1H, NH_exch_), 7.83–7.43 (m, 9H, H_arom_ + NH), Anal. for C_13_H_11_N_3_O_3_S (289.30):Calcd/found C:53.97/53.95, H: 3.83/3.81 and N:14.52/14.50%.

### *N*-[5-(Cyanomethyl)-1,3,4-oxadiazol-2-yl]furan-2-carboxamide (**a4**)

Brown powder (80% yield), mp > 300 °C. IR (ν^–^, cm^–1^): 3131 (NH), 3006 (CH_arom_), 2970 (CH_aliph_), 2200 (CN), 1660 (C=O) (Figure S4); ^1^H NMR (DMSO-*d*_6_), (δ, ppm): 13.17 (s, 1H, NH), 8.08 (s, 1H, CH_arom_), 7.75 (s, 1H, CH_arom_), 6.78 (s, 1H, H_arom_), 4.61 (s, 2H, CH_2aliph_) (Figure S5). ^13^C NMR: 184.53, 174.15, 171.15, 163.35, 147.61, 119.15, 116.36, 116.63, 113.03, 116.46 (Figure S6). Anal. for C_9_H_6_N_4_O_3_ (218.169) Calcd/found: C: 49.55/49.50, H: 2.77/2.70, and N: 25.68/25.60%.

### *N*-(5-Phenyl-1,3,4-oxadiazol-2-yl)furan-2-carboxamide (**a5**)

White solid (80% yield), mp. 240–242 °C. IR (ν^–^, cm^–1^): 3102.60 (NH), 3016.63 (CH_arom_), 1675.55 (C=O) (Figure S7); ^1^H NMR (DMSO-*d*_6_), (δ, ppm): 13.10 (s, 1H, NH_exch_), 8.04–6.76 (m, 8H, H_arom_) (Figure S8). ^13^C NMR: 162.42, 159.27, 156.32, 148.18, 145.82, 131.07, 130.61, 129.80, 127.41, 117.92, 112.91 (Figure S9). Anal. for C_13_H_9_N_3_O_3_ (255.22) Calcd/found: C: 61.18/61.17, H: 3.55/3.54, and N: 16.46/16.45%.

### General Procedure for the Synthesis of Compounds **b2–5**

While stirring an equimolecular of ammonium thiocyanate (50 mmol) in 20 mL of dry acetone, an acid chloride named 2,6-dichlorobenzoyl chloride or 4-fluorobenzoyl chloride in 15 mL of acetone (50 mmol) was added dropwise and refluxed for 3 h at 200 °C. The amino derivative [4-chlorobenzohydrazide, 1-(4-aminophenyl)ethanone, and cyanoacetohydrazide)] (30 mmol) in 15 mL of acetone was added to the mixture, and then the reaction mixture was refluxed for 3 h. The precipitate was collected and washed thoroughly with H_2_O and crystallized from a methanol/dichloromethane mixture (1:1).

### 2,6-Dichloro-*N*-{[2-(4-chlorobenzoyl)hydrazinyl]carbonothioyl}benzamide (**b2**)

Yellow solid (80% yield) mp > 300 °C; IR (ν^–^, cm^–1^): 3151 (NH), 3008.30 (CH_arom_), 1688.97(C=O), 1607.50 (C=C). ^1^H NMR (DMSO-*d*_6_), (δ ppm): 11.89 (s, 1H, NH_exch_), 11.24 (s, 1H, NH_exch_), 9.90 (s, 1H, NH_exch_), 7.36–8.80 (m, 7H, H_arom_) (Figure S11). ^13^C NMR: 181.34, 169.71, 168.64, 163.73, 152.34, 151.34, 131.43, 130.39, 128.92, 128.62, 116.64, 115.64 (Figure S8). Anal. for C_15_H_10_Cl_3_N_3_O_2_S (402.68): Calcd./found: C:44.74/44.73,H: 2.50/2.49 and N:10.44/10.43%.

### *N*-[(4-Acetylphenyl)carbamothioyl]-2,6-dichlorobenzamide (**b3**)

Brown solid (85% yield), mp 140–141 °C; IR (ν^–^, cm^–1^): 3312.57 (NH), 3210.44 (NH), 3060.99 (CH_arom_), 1667.21 (C= O). ^1^H NMR (DMSO-*d*_6_), (δ ppm): 12.41 (s, 1H, NH_exch_), 11.13 (s, 1H, NH_exch_), 7.58–8.01 (s, 7H, H_arom_), 2.61 (s, 3H, CH_3_) (Figure S12). ^13^C NMR: 194.13, 181.12, 156.13, 163.73, 154.56, 149.12, 142.56, 139.14, 131.49, 126.13, 124.16, 115.24, 31.12 (Figure S13). Anal. for C_16_H_12_Cl_2_N_2_O_2_S (367.24): Calcd./found: C, 52.33/52.30, H:3.92/3.90, and N:7.63/7.61%.

### *N*-[(4-Acetylphenyl)carbamothioyl]-4-fluorobenzamide (**b4**)

White powder (76% yield), mp 200–201 °C. IR (ν^–^, cm^–1^): 3473–3102 (NH), 3060 (CH_arom_), 1676 (C=O) (Figure S14); ^1^H NMR (DMSO-*d*_6_), (δ, ppm): 12.67 (s, 1H, NH), 11.76 (s, 1H, NH), 7.34–8.08 (m, 8H, H_arom_), 2.56 (s, 3H, CH_3_) (Figure S15). ^13^C NMR: 197.30, 173.44, 169.34, 166.34 166.30, 164.13, 142.62, 136.71, 135.11, 134.11, 130.12, 118.11, 116.64, 29.14 (Figure S16). Anal. for C_16_H_13_FN_2_O_2_S (3.16.35) Calcd/found: C: 60.75/60.72, H: 4.14/4.11, and N: 8.86/8.85%.

### *N*-{[2-(Cyanoacetyl)hydrazinyl]carbonothioyl}-4-fluorobenzamide (**b5**)

Yellow powder (80% yield), mp 195–196 °C. IR (ν^–^, cm^–1^): 3300–3216 (3NH), 3051 (CH_arom_), 2937 (CH_aliph_), 2200 (CN), 1679 (C=O) (Figure S17); ^1^H NMR (DMSO-*d*_6_), (δ, ppm): 12.82 (s, 1H, NH), 12.64 (s, 1H, NH), 11.29 (s, 1, NH), 8.07–7.34 (m, 4H, H_arom_), 3.96 (s, 2H, CH_2aliph_) (Figure S18). ^13^C NMR: 180.12, 169.37, 161.13, 158.50, 151.80, 136.12, 132.30, 113.14, 23.64 (Figure S19): Anal. for C_11_H_9_FN_4_O_2_S (280.27) Calcd/found: C: 47.14/47.10, H: 3.24/3.20 and N: 19.99/19.89%.

### General Procedure for the Synthesis of Compounds **c2–5**

An equimolecular amount (5 mmol) of cyanoacetohydrazide or 4-aminoacetophenone in 15 mL of acetone and phenylisocyanate (**c1**) (5 mmol) was added and refluxed for 3 h. The precipitate was collected and washed thoroughly with H_2_O and crystallized from a methanol/dichloromethane mixture (1:1) to produce **c2** and **c5**, respectively.

An equimolecular amount (5 mmol) of compound **c2** in ethanol (15 mL) was added to an appropriate reagent, namely, hydrazine or phenylhydrazine, and the reaction mixture was refluxed for 5 h. The precipitate was collected by filtration, washed thoroughly with H_2_O, dried, and purified by crystallization from ethanol to produce **c4** and **c3**, respectively. On the other hand, a suspension of compound **c2** (10 mmol) in chloroform (20 mL) and cyanoguanidine (3 mmol) was added. The reaction mixture was refluxed for 3 h and cooled to room temperature, and the precipitate was collected by filtration, washed thoroughly with H_2_O, dried, and purified by crystallization from ethanol to give compound **c6.**

### 2-[Cyanoacetyl]-*N*-phenylhydrazine-2-carboxamide (**c2**)

White solid crystal yield (89%); mp 160–161 °C; IR (ν^–^, cm^–1^): 3329.71 (NH), 3307.28 (NH), 3210.63 (NH), 3017.88 (CH_arom_), 2958.63 (CH_aliph_), 2200 (CN), 1708.18 (C=O), 1661.81 (C=O), 1608.45 (C=C) (Figure S20). ^1^H NMR (DMSO-*d*_6_), (δ ppm): 10.00 (s, 1H, NH_exch_), 8.70 (s, 1H, NH_exch_), 8.20 (s,1H, NH), 7.45–6.98 (m, 4H, H_arom_), 3.72 (s, 2H, CH_2alpht_) (Figure S21). ^13^C NMR: 162.62, 155.39, 129.08, 122.29, 119.28, 116.06, 26.37 (Figure S22). Anal. for C_10_H_10_N_4_O_2_ (218.212): Calcd/found; C: 55.04/55.02, H: 4.62/4.61, and N: 25.68/25.66%.

### 2-(5-Imino-4,5-dihydro-1*H*-pyrazol-3-yl)-*N*-phenylhydrazinecarboxamide (**c3**)

Brown solid (80% yield), mp 140–141 °C. IR (ν^–^, cm^–1^): 3408.59 (NH), 3261.51 (NH), 3031.43 (CH_arom_), 1647.78 (C=O), 1592.53 (C=C) (Figure S23); ^1^H NMR (DMSO-*d*_6_), (δ, ppm): 9.71 (s, 1H, NH_exch_), 8.64 (s, 1H, NH_exch_), 8.60 (s, 1H, NH_exch_), 7.51–6.91 (m, 5H, CH_arom_ + 2NH), 4.36(s, 2H, CH_2__aliph_) (Figure S24). ^13^C NMR: 157.82, 142.82, 140.37, 140.15, 133.44, 129.23, 129.01, 122.31, 121.85, 118.71, 118.61, 39.60 (Figure S25). Anal. for C_10_H_12_N_6_O (232.24) Calcd/found: C: 51.72/51.71, H: 5.21/5.19, and N: 36.19/36.18%.

### 2-(5-Imino-1-phenyl-4,5-dihydro-1*H*-pyrazol3-yl)-*N*-phenylhydrazinecarboxamide (**c4**)

Red solid (70% yield); mp. 190–192 °C. IR (ν^–^, cm^–1^): 3449.60 (NH), 3330 (NH), 3212.22 (NH), 3022.5 (CH_arom_), 1659.42 (C=O), 1592.53 (C=C). ^1^H NMR (DMSO-*d*_6_), (δ ppm): 10.03 (s, 1H, NH), 8.76 (s, 1H, NH), 8.64–6.98 (m, 10H, CH_arom_), 6.81 (s, 1H, NH), 3.73 (s, 2H, CH_2aliph_), 3.35 (s, 1H, NH_ach_), ^13^C NMR: 163.05(C=O), 157.04 (C–NH), other aromatic C–H carbons at 156.52, 155.46, 154.07, 149.78, 140.09, 139.78, 138.53, 134.05, 130.76, 130.17, 129.94, 129.51, 129.24, 129.11, 128.97, 124.66, 124.32, 123.60, 122.68, 122.34, 121.43, 120.05, 119.56, 119.31, 118.99, 118.75, 116.08, 113.01, 24.36 (CH_2aliph_). Anal. for C_16_H_16_N_6_O (308.337) Calcd/found: C:62.32/62.3, H:5.23/5.19, and N:27.26/27.56%.

### 1-(4-Acetylphenyl)-3-phenylurea (**c5**)

Yellow solid (86% Yield) mp. 180–182 °C; IR (ν^–^, cm^–1^): 3341.80 (NH), 3304.45 (NH), 3040.48 (CH_arom_), 1740.28 (C=O), 1655.54 (C=O).^1^H NMR (DMSO-*d*_6_), (δ ppm): 9.04 (s, 1H, NH), 8.74 (s, 1H, NH_exch_), 7.92–7.01 (m, 9H, H_arom_), 2.52 (s, 3H, CH_3_) (Figure S26). ^13^C NMR: 197.85, 152.82, 144.66, 139.31, 130.96, 130.13, 129.33, 123.11, 119.28, 117.87, 26.63 (Figure S27). Anal. For C_15_H_14_N_2_O_2_ (254.28): Calcd./found C: 70.85/70.84, H: 5.55/5.53, and N: 11.02/11.01%.

### 1-{4-[(6-Amino-1-phenyl-1,4-dihydro-1,3,5-triazinyl)amino]-phenyl}ethanone (**c6**)

Brown crystal (80% yield), mp 209–210 °C. IR (ν^–^, cm^–1^): 3476.77, 3386.62 (NH_2_, NH), 3243.09 (NH), 3188.03 (NH), 3005.83 (CH_arom_), 2898.16 (CH_aliph_), 1610.19(C=O), 1596.98 (C=C) (Figure S28); ^1^H NMR (DMSO-*d*_6_), (δ, ppm): 9.12 (s, 2H, 2NH), 7.89–6.97 (m, 9H, H_arom_ + s, 3H, NH), 2.51 (s, 3H, CH_3aliph_) (Figure S29). ^13^C NMR: 196.70, 152.81, 145.04, 139.94, 130.83, 130.03, 129.21, 122.57, 118.97, 117.64, 26.72 (Figure S30). Anal. For C_17_H_16_N_6_O (320.34) Calcd/found: C: 63.74/63.73, H: 5.03/5.02, and N: 26.23/26.21%.

Compounds **a2–5**, **b2–5**, **c3**, **c4**, and **c6** were tested against the second larval insect. As shown in Table 1, the bioefficacy results of tested compounds exhibit high to low toxicological activity against the second larvae, for which the LC_50_ values vary from 26.63 to 313.11 ppm; for example, the LC_50_ values of compounds **a2–5**, **b2–5**, **c3**, **c4**, and **c6** were 97.01, 114.10, 73.35, 186.48, 46.35, 60.84, 86.93, 26.63, 128.41, 145.56, and 313.11 ppm, respectively, and that of the lufenuron standard insecticide value was 17.01 ppm. Thus, the toxicity of synthesized compound **b5** against the second larva instar insect was close to the insecticidal bioactivity of reference lufenuron.

**Table 1:** Insecticidal Activity of Compounds **a2–5**, **b2–5**, **c3**, **c4**, **c6**, and Lufenuron as the Reference Insecticide against the Second and Fourth Larva Instar of *S. littoralis* after 72 h of Treatment

Second instar larvae				4th instar larvae		
comp.	LC_50_ (ppm)	slope	toxic ratio^a^	LC_50_ (ppm)	slope	toxic ratio^a^
Lufenuron	17.01	0.246 ± 0.0791	1	103.12	0.234 ± 0.083	1
**a2**	97.37	0.229 ± 0.0813	0.174	798.35	0.418 ± 0.953	0.129
**a3**	114.10	0.307 ± 0.0993	0.147	881.36	0.853 ± 0.287	0.117
**a4**	73.35	0.460 ± 0.0805	0.231	793.35	0.418 ± 0.953	0.129
**a5**	186.48	1.365 ± 0.388	0.279	965.18	2.083 ± 1.0779	0.106
**b2**	46.35	1.295 ± 0.3923	0.366	148.56	0.302 ± 0.0953	0.694
**b3**	60.84	0.225 ± 0.8201	0.279	152.06	0.239 ± 0.0985	0.677
**b4**	86.93	0.231 ± 0.0823	0.196	745.39	3.176 ± 1.184	0.137
**b5**	26.63	0.246 ± 0.0805	0.638	145.90	0.307 ± 0.0993	0.706
**c3**	128.41	0.2971 ± 0.0978	0.133	254.41	0.225 ± 0.0820	0.405
**c4**	145.56	0.307 ± 0.0993	0.116	1253.1	0.422 ± 0.938	0.082
**c6**	313.11	1.815 ± 1.0141	0.0543	1588.3	0.4.36 ± 1180	0.064

a Notes: toxicity ratio is calculated as lufenuron’s LC_50_ value for baseline toxicity/the compounds’ LC_50_ value. Toxicological activity test for the second larvae.

Table 1 shows the result of toxicity for the synthesized compounds **a2**–**5**, **b2**–**5**, **c3**, **c4**, and **c6**. After 72 h of treatment, high to low toxicological activity against the fourth larvae was observed, for which the LC_50_ values vary from 145.90 to 1588.36 ppm; for example, the LC_50_ values of compounds **a2–5**, **b2–5**, **c3**, **c4**, and **c6** were 798.35, 881.36, 793.35, 965.18, 148.56, 152.06, 745.39, 145.90, 254.41, 1253.1, and 1588.3 ppm, respectively. The results obtained for compounds **a2–5**, **b2–5**, **c3**, **c4**, and **c6** showed that **b5** > **b2** > **b3** > **a4** > **b4** > **a2** > **c3** > **a3** > **a5** > **c4** > **c6**, for which the lufenuron value was 103.12 ppm. For this result, the toxicity of compound **b5** and **b2** against 4th larvae was nearly lufenuron after 72 h. The LC_50_ value of compounds **b5** and **b2** was 145.90 and 148.56 ppm, respectively, and that of lufenuron was 103.12 ppm.

### Structure–Activity Relationship

**Figure 1:**
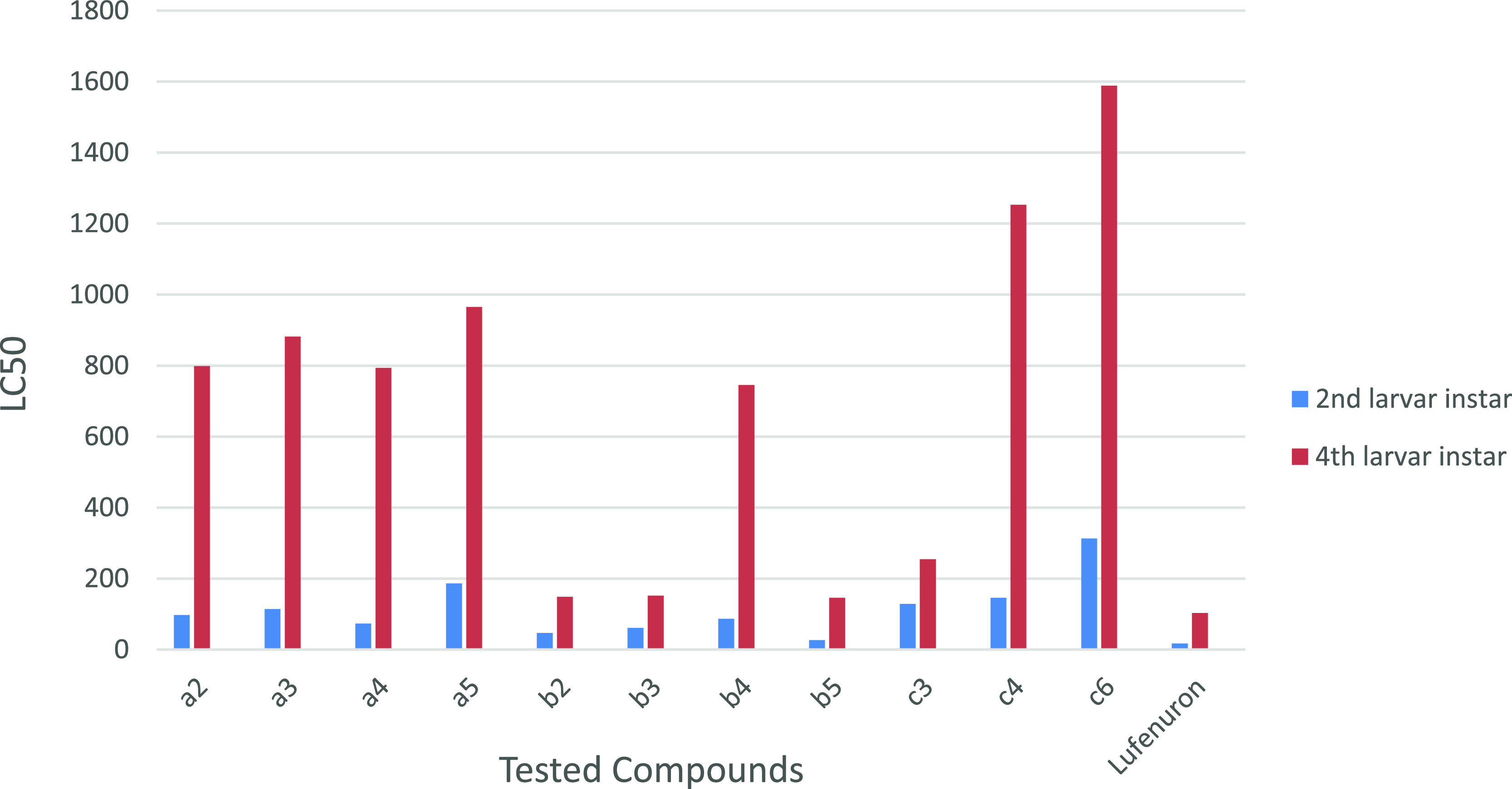
Insecticidal activity of compounds **a2**–**5**, **b2**–**5**, **c3**, **c4**, and **c6** against the second and fourth larva instar of *S. littoralis* (Boisd.) after 72 h of treatment compared to lufenuron as the standard insecticide. Toxicological activity test for adult fourth larvae.

**Figure 2:**
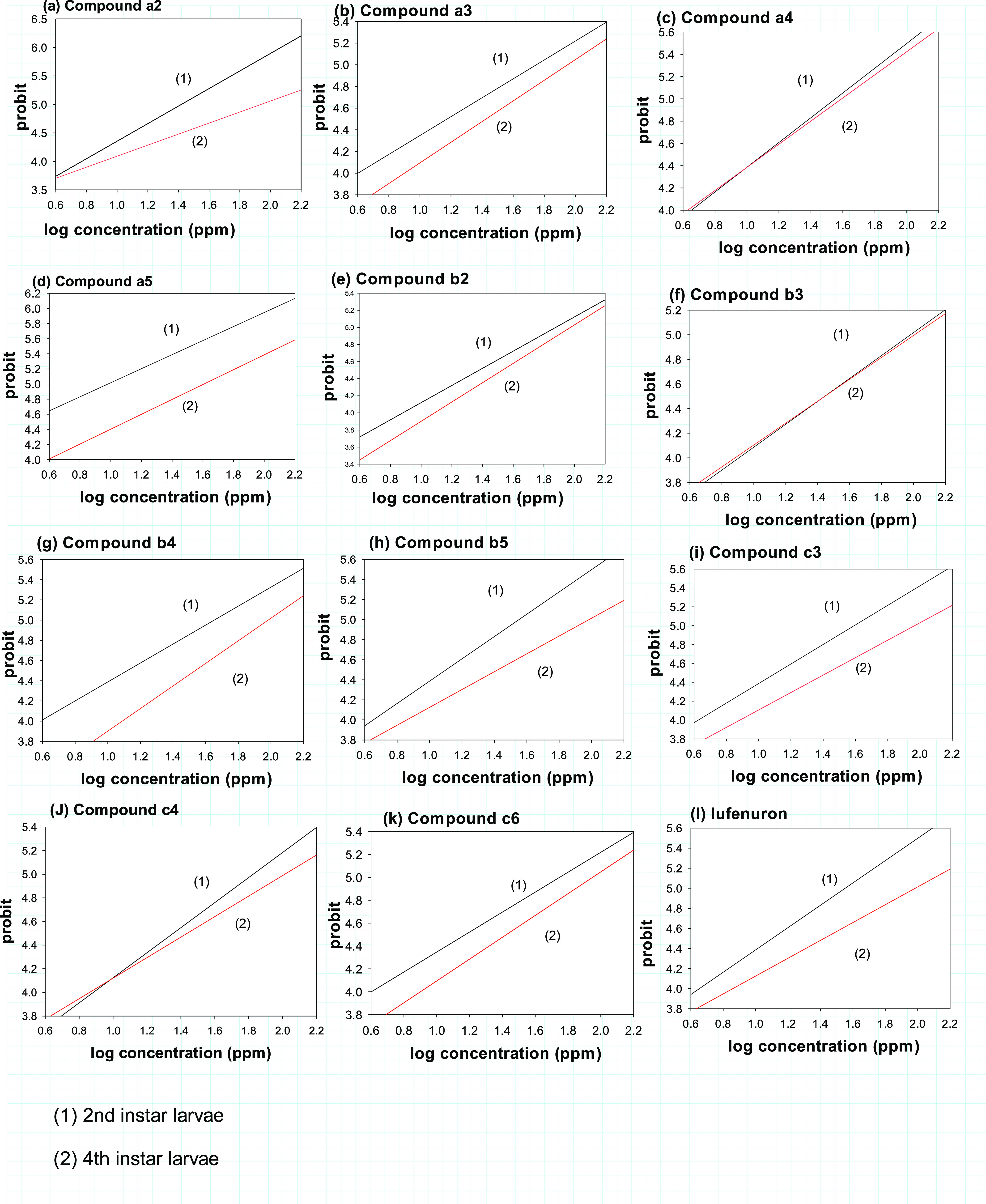
Insecticidal activities of selective compounds **a2–5**, **b2–5**, **c3**, **c4**, **c6**, and lufenuron as the reference insecticide for the second and fourth larval instar of *S. littoralis* after treatment.

According to the toxicity values in Table 1 and Figure 1, by using a computerized regression analysis program, the median lethal concentration (LC_50_) and slope values of the target compounds were computed and reported as parts per million (ppm). The insecticidal activity of the synthesized compounds (**a2–5**, **b2–5**, **c3**, **c4**, and **c6**) was compared with that of lufenuron against *S. littoralis*, for which the second instar larvae are represented by black lines and the fourth instar larvae are represented by red lines after 72 h of treatment (Figures 2 and S32). The structure–activity relationship was established. The benzamide compound **b5** is more active against the second and fourth larval insects than the other benzamide-synthesized derivatives. The high activity of compound **b5** may be due to the presence of fluorophenyl and cyano in its structure. The presence of fluorophenyl and cyano moieties in this compound which is considered as an electron-withdrawing group increases the activity of the other urea and/or thiourea-synthesized derivatives compared to the commercial lufenuron insecticide. On the other hand, compound **b4** exhibited good activity, which might be due to the presence of the fluorophenyl moiety in its structure. Also, the toxicity of compound **b2** was higher, which might be due to the presence of a dichlorophenyl group in its structure. The aromatic moiety also enhanced the insecticidal activity. The **b5** compound is higher in toxicity than compounds **b2**, **b3**, and **a4**, and this is due to the presence of fluorophenyl and cyano groups in its structure.

## Conclusions

A new series of urea, thiourea, thiosemicarbazide, pyrazole, oxadiazole, and triazine derivatives have been prepared in a good yield via the reaction of an acid chloride and an equimolar amount of ammonium thiocyanate in dry acetone and amine derivatives, and their chemical structure was established based on spectral and elemental data. The synthesized compounds are analogous to insect growth-regulating insecticides. The activity of these new compounds was tested against the second and fourth larval insects, and they showed good toxicological activities. It has been found that the compound **b5** has an activity close to that of the standard reference lufenuron, whose LC_50_ was found to be 26.63 ppm, whereas the LC_50_ for lufenuron was 17.01 ppm, which might be due to the presence of a fluorine atom and a cyano group in its structure. In the structure–activity relationship studies, the synthesized compounds that contain a halogen in their structure play a principal role in the activity..

## Supporting Information Available

IR spectra, ^1^H NMR spectra, ^13^C NMR spectra, and elemental analysis of urea, thiourea, thiosemicarbazide, oxadiazole, pyrazole, and triazine derivatives (PDF)
