# Supplementary material for: Design, Synthesis, and Toxicological Activities of Novel Insect Growth Regulators as Insecticidal Agents against Spodoptera littoralis (Boisd.)
Source: ACS Omega. 2022 Dec 20;8(1):709–17. doi: 10.1021/acsomega.2c05977 (PMC9835546; doi:10.1021/acsomega.2c05977)
Supplement: Supplementary file 1 — ao2c05977_si_001.pdf [file ao2c05977_si_001.pdf]

Supporting information

Design, synthesis and toxicological activities of novel insect growth regulators as insecticidal agents against *Spodoptera littoralis* (Boisd.)

Antar A. Abdelhamid[a,b], Safwat A. Aref[c], Nabila A. Ahmed[c], Ahmed M. M. Elsaghier[a], Fawy M. Abd El Latif[b,d], Sameera N. Al-Ghamdi[b], Mohamed A. Gad*[c]

[a] Department of Chemistry, Faculty of Science, Sohag University, 8252, Sohag, Egypt.

[b] Chemistry Department, Faculty of Science, Albaha University, Albaha 1988, Saudi Arabia

[c] Research Institute of Plant Protection, Agricultural Research Center, 12112 Giza, Egypt.

[d] Chemistry Department, Faculty of Science, Aswan University, Aswan 81528, Egypt

List of Figures:

Figure (S1): IR spectrum for compound a2

Figure (S2): ^{1}HNMR spectrum for compound a2

Figure (S3): ^{13}CNMR spectrum for compound a2

Figure (S4): IR spectrum for compound a4

Figure (S5): ^{1}HNMR spectrum for compound 4a

Figure (S6): ^{13}CNMR spectrum for compound 4a

Figure (S7): IR spectrum for compound a5

Figure (S8): ^{1}HNMR spectrum for compound 5a

Figure (S9): ^{13}CNMR spectrum for compound 5a

Figure (S10): ^{1}HNMR spectrum of compound b2

Figure (S10): ^{13}CNMR spectrum of compound b2

Figure (S12): ^{1}HNMR spectrum of compound b3

Figure (S113): ^{13}CNMR spectrum of compound b3

Figure (S14): IR spectrum of compound b4

Figure (S15): ^{1}HNMR spectrum of compound b4

Figure (S16): ^{13}CNMR spectrum of compound b4

Figure (S17): IR spectrum of compound b5

Figure (S18): ^{1}HNMR spectrum of compound b5

Figure (S19): ^{13}CNMR spectrum of compound b5

Figure (S20): IR spectrum for compound c2

Figure (S21): ^{1}HNMR spectrum for compound c2

Figure (S22): ^{13}CNMR spectrum for compound c2

Figure (S23): ^{1}HNMR spectrum for compound c3

Figure (S24): ^{1}HNMR spectrum for compound c3

Figure (S125): ^{13}CNMR spectrum for compound c3

Figure (S26): ^{1}HNMR spectrum for compound c5

Figure (S27): ^{13}CNMR spectrum for compound c5

Figure (S28): IR spectrum for compound c6

Figure (S29): ^{1}HNMR spectrum for compound c6

Figure (S30): ^{13}CNMR spectrum for compound c6

Figure (S31): Insecticidal activity of compounds a2-5, b2-5, c3, c4 and c6 against the 2nd and 4th larvae instar of *S. littoralis* after 72 hours of treatment compared to Lufenuron as the standard insecticide.

Figure (S32): Insecticidal activities of selective compounds a2-5, b2-5, c3, c4, c6 and Lufenuron as reference insecticide for the 2nd and 4th larvae instar of *S. littoralis* after treatment.

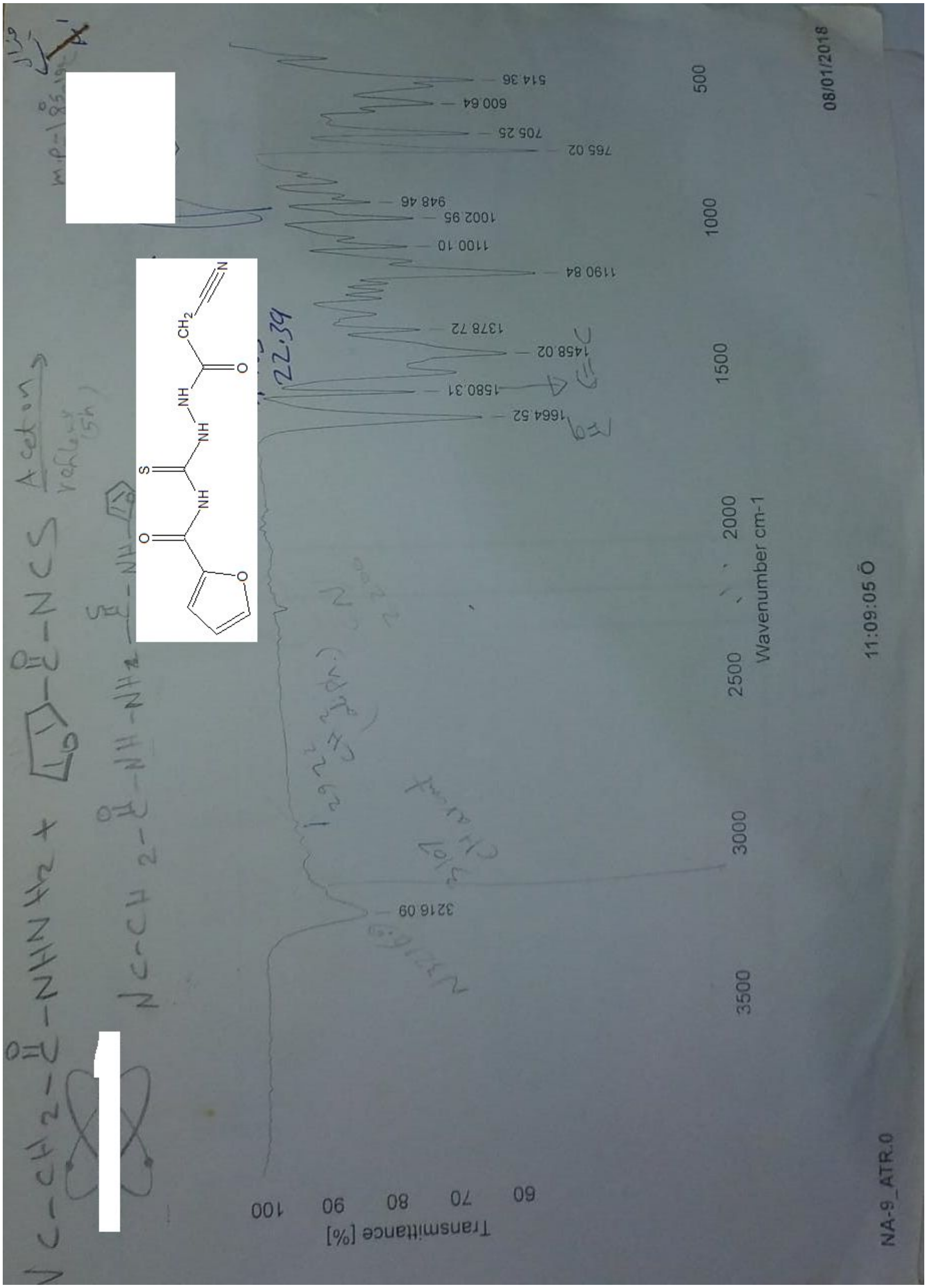

Figure (S1): IR spectrum for compound a2

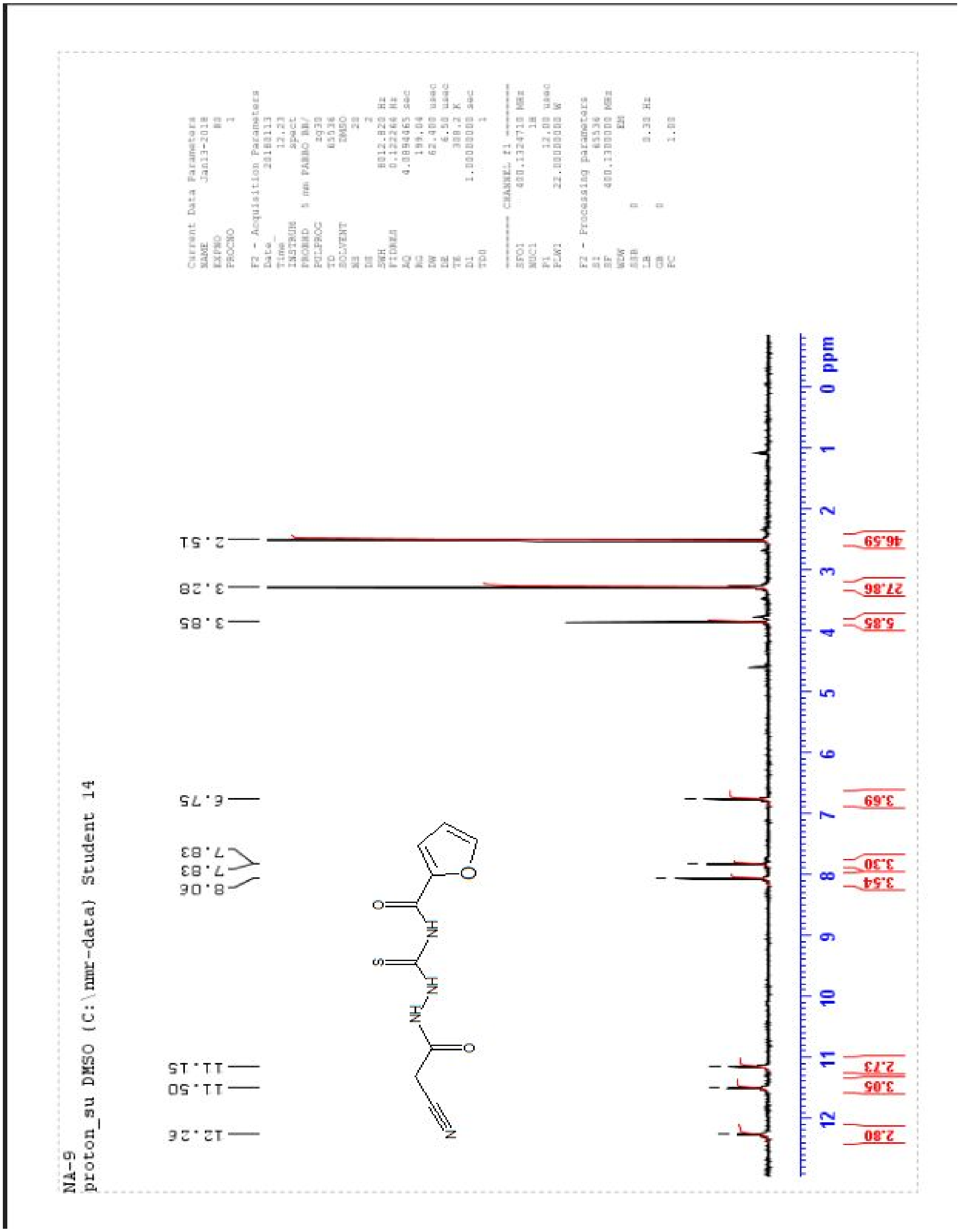

Figure (S2): ^{1}HNMR spectrum for compound a2

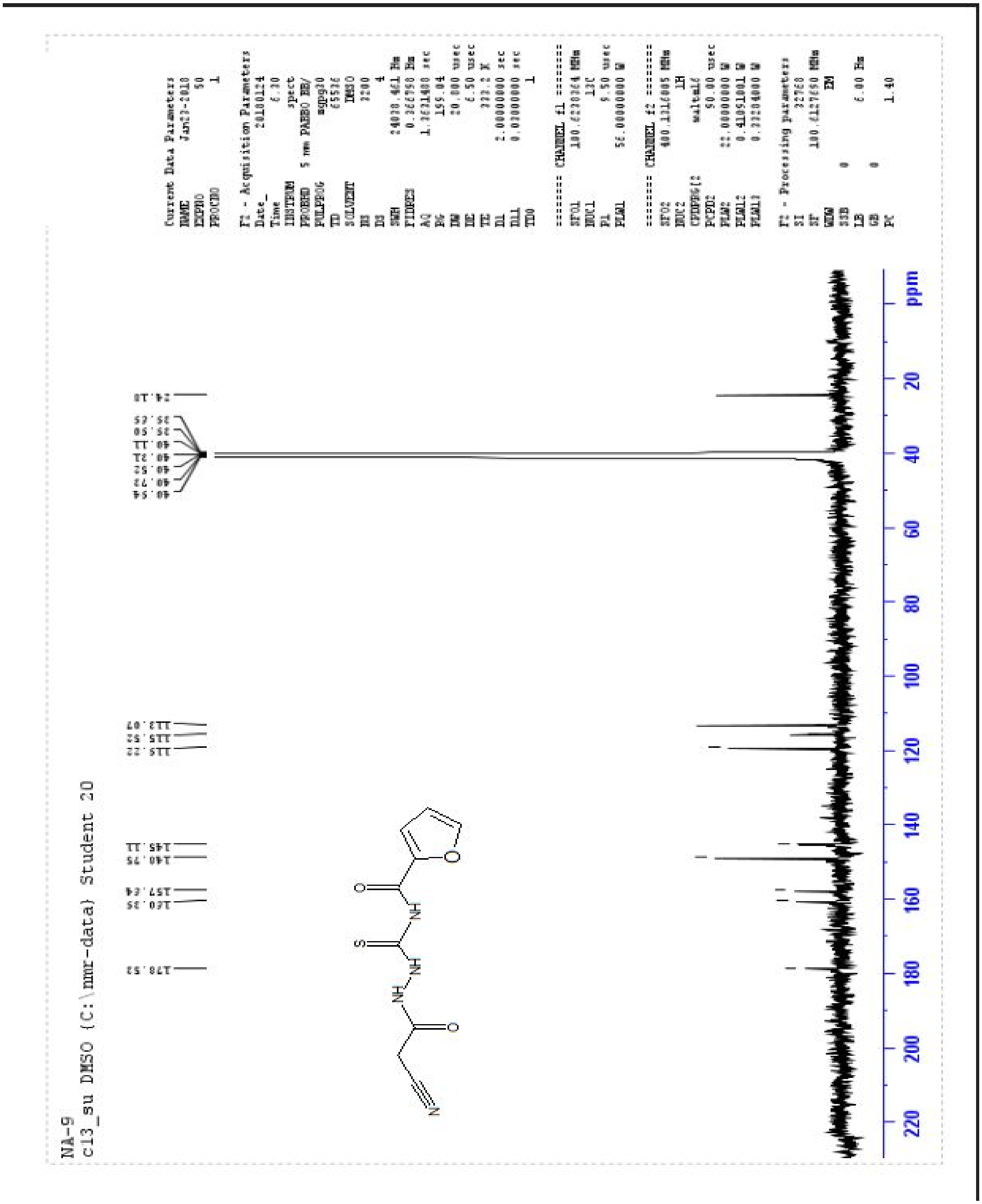

Figure (S3): ^{13}CNMR spectrum for compound a2

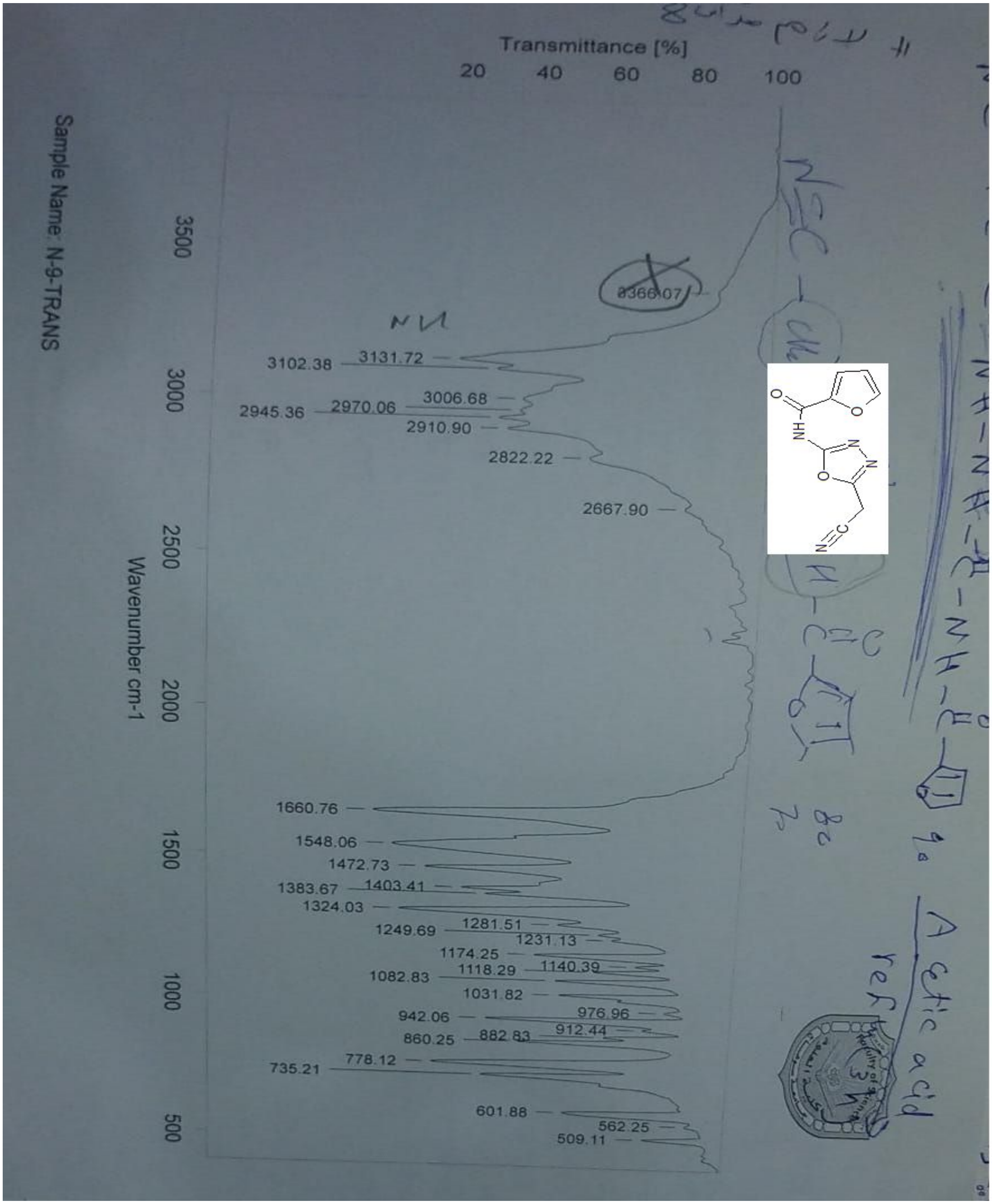

Figure (S4): IR spectrum for compound a4.

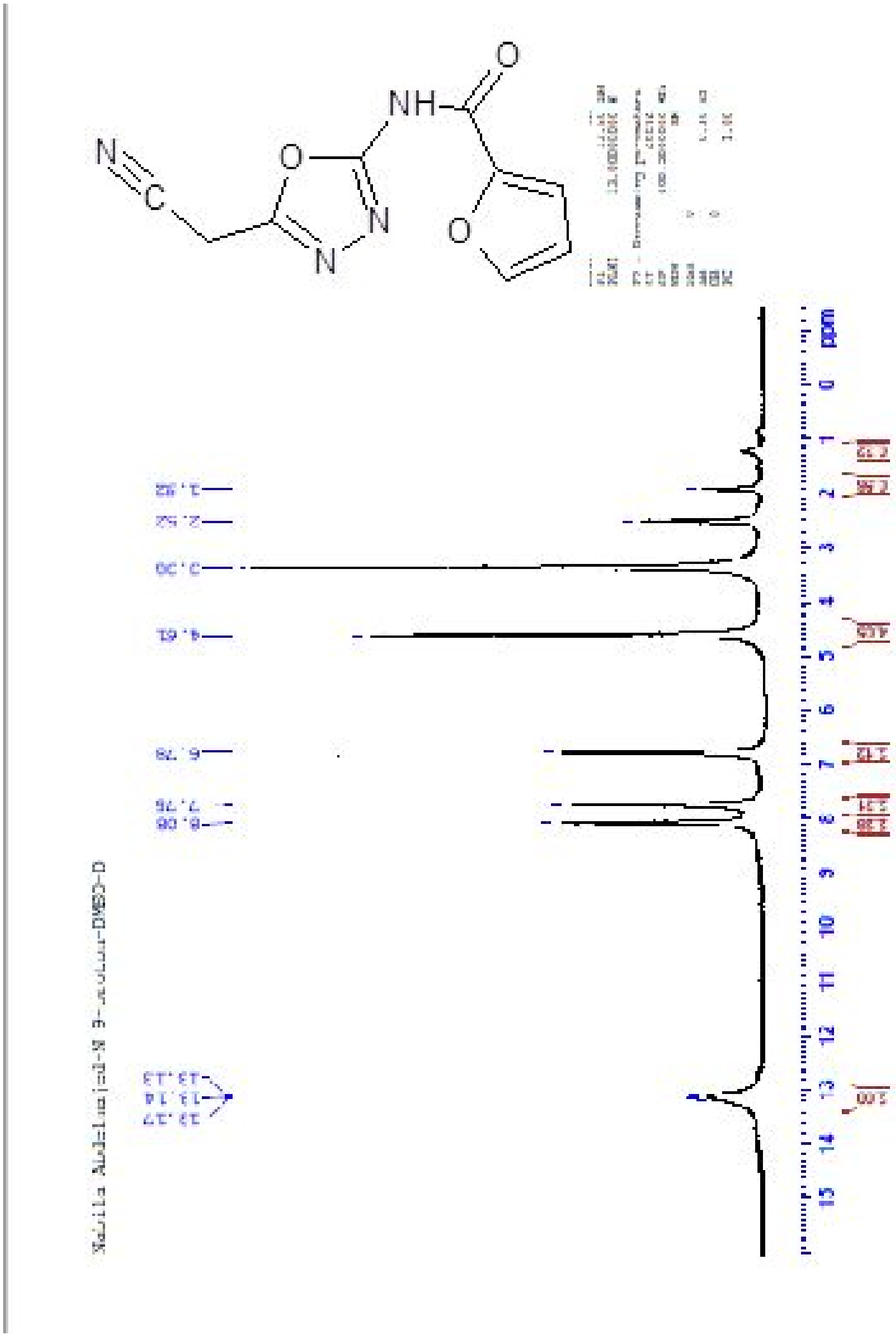

Figure (S5): ^{1}HNMR spectrum for compound a4

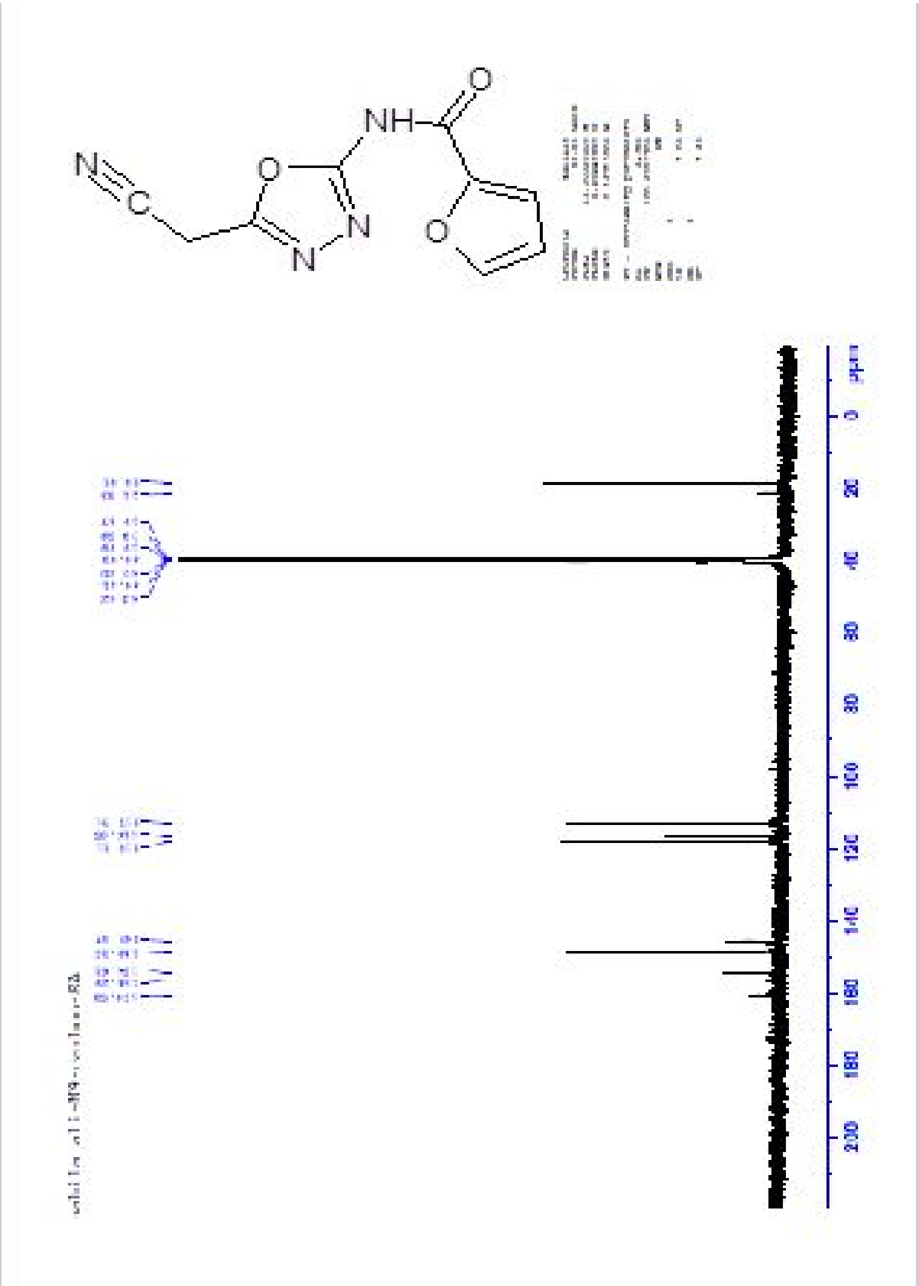

Figure (S6): ^{13}CNMR spectrum for compound a4

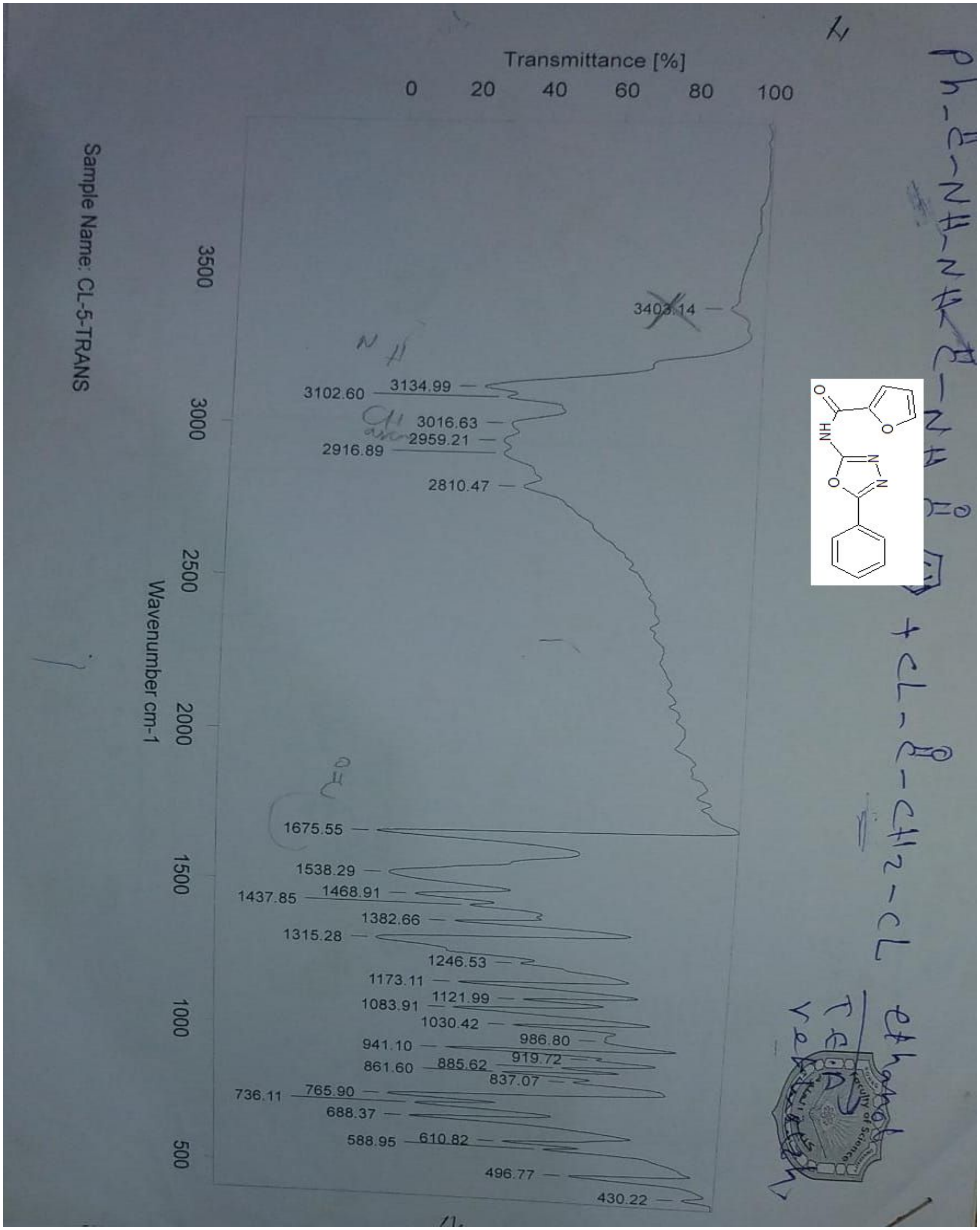

Figure (S7): IR spectrum for compound a5

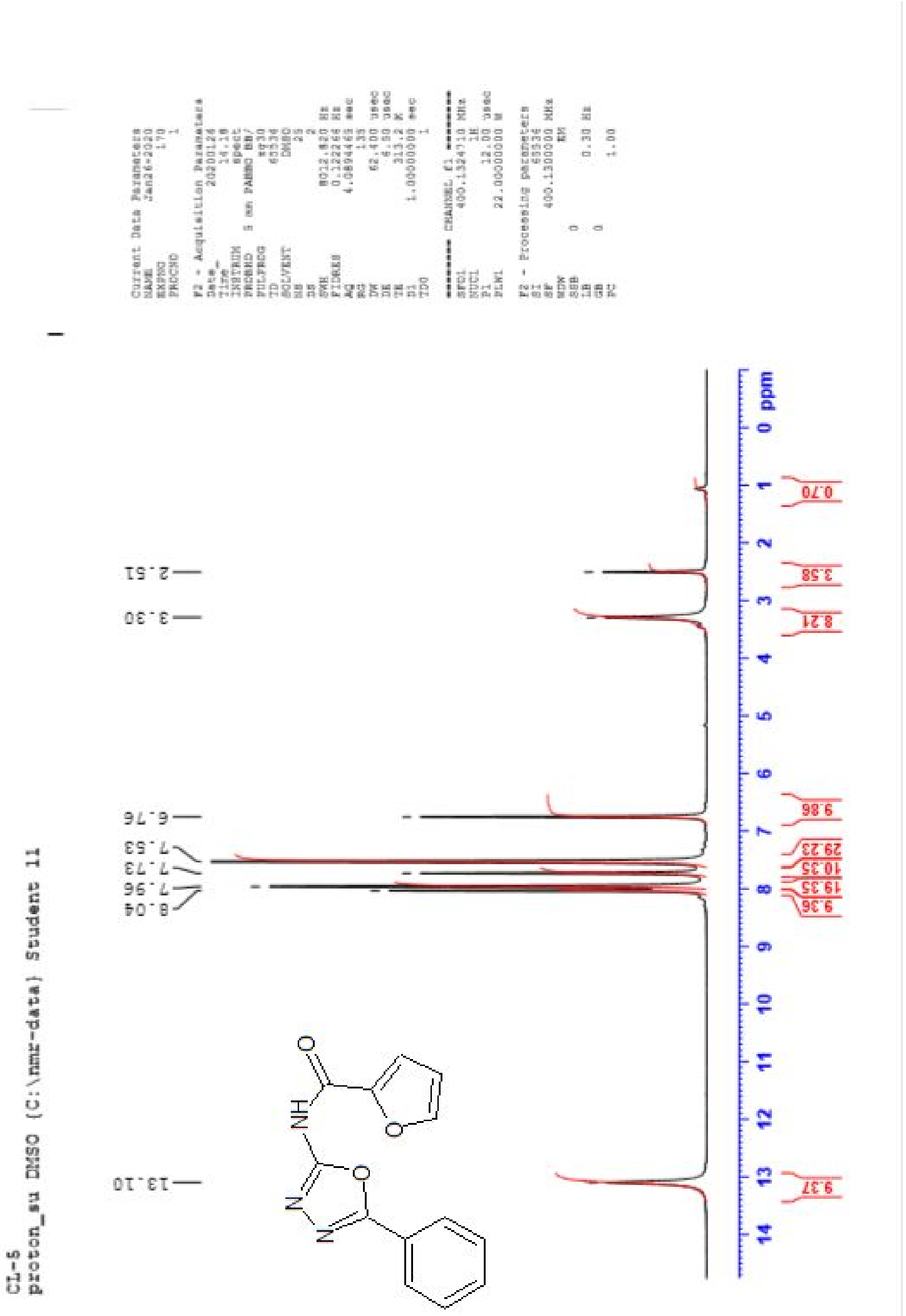

Figure (S8): ^{1}HNMR spectrum for compound a5

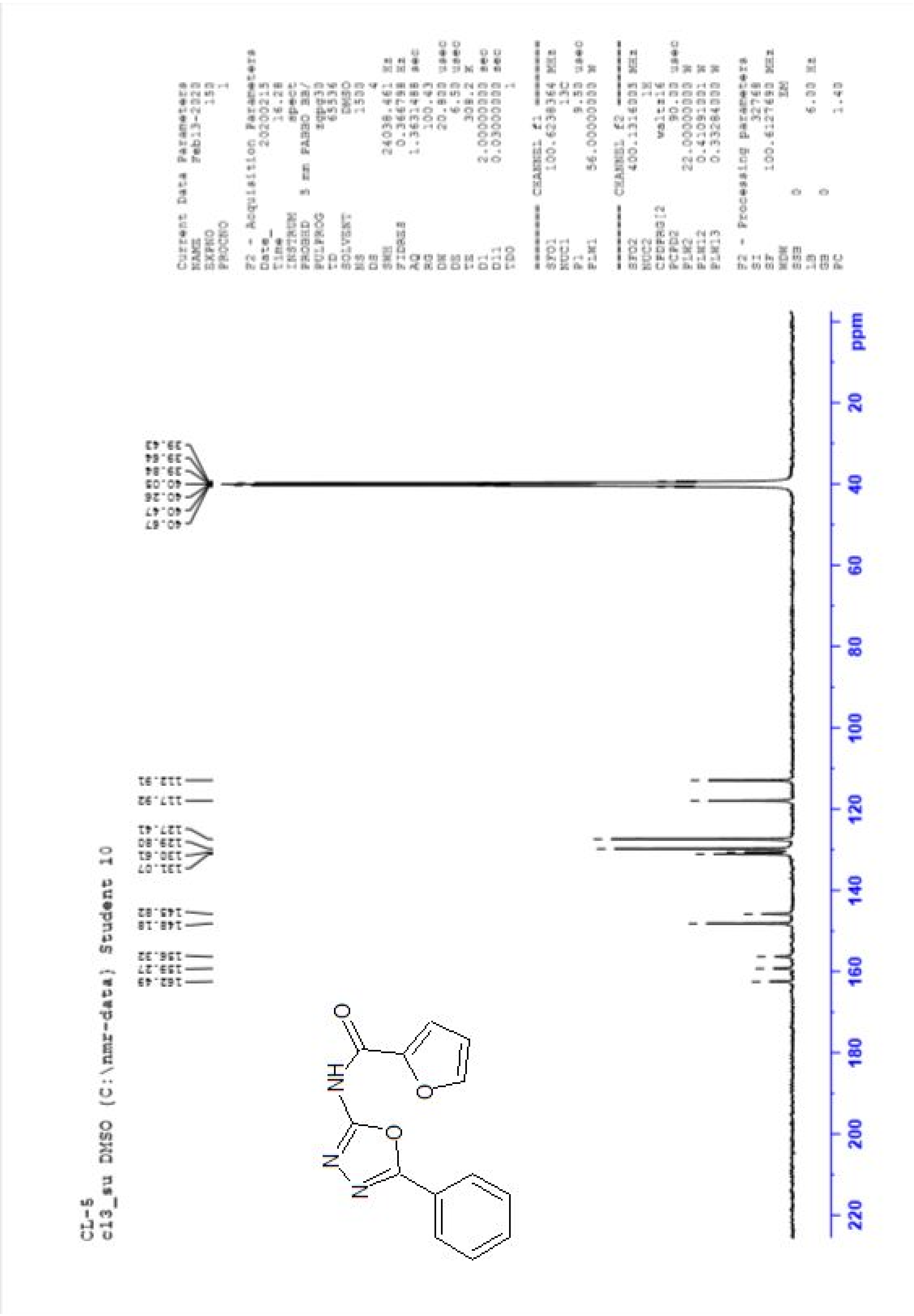

Figure (S9): ^{13}CNMR spectrum for compound 5a

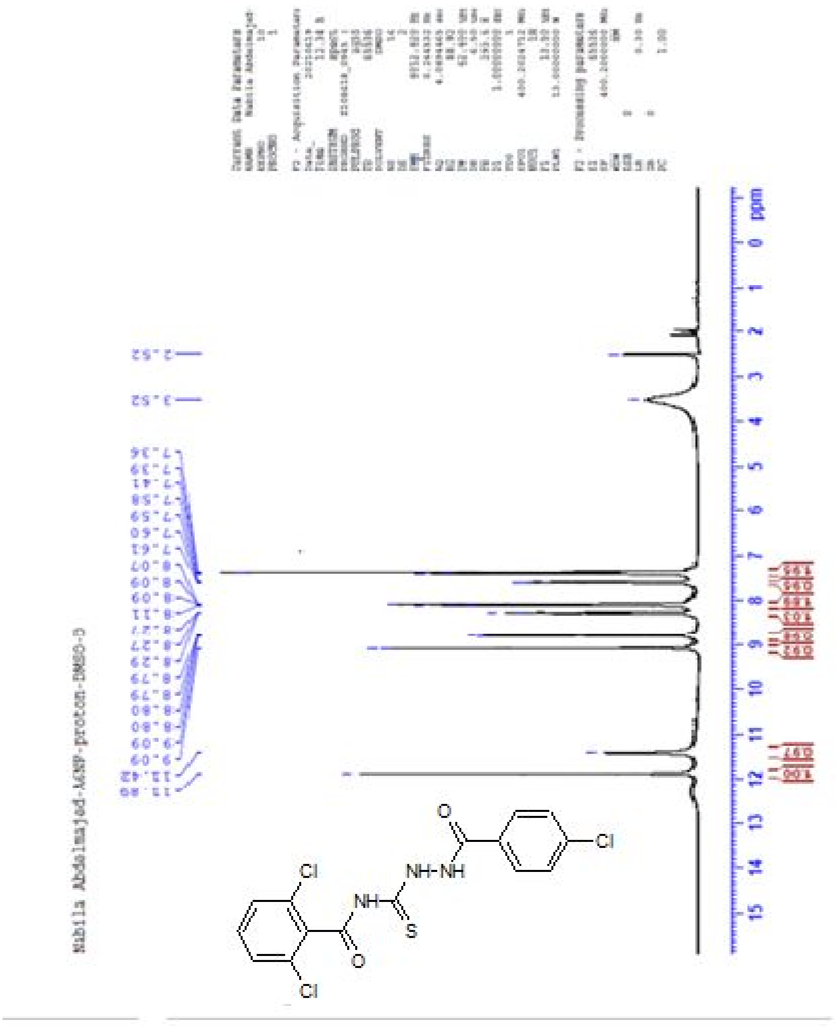

Figure (S10): ^{1}HNMR spectrum of compound **b2**

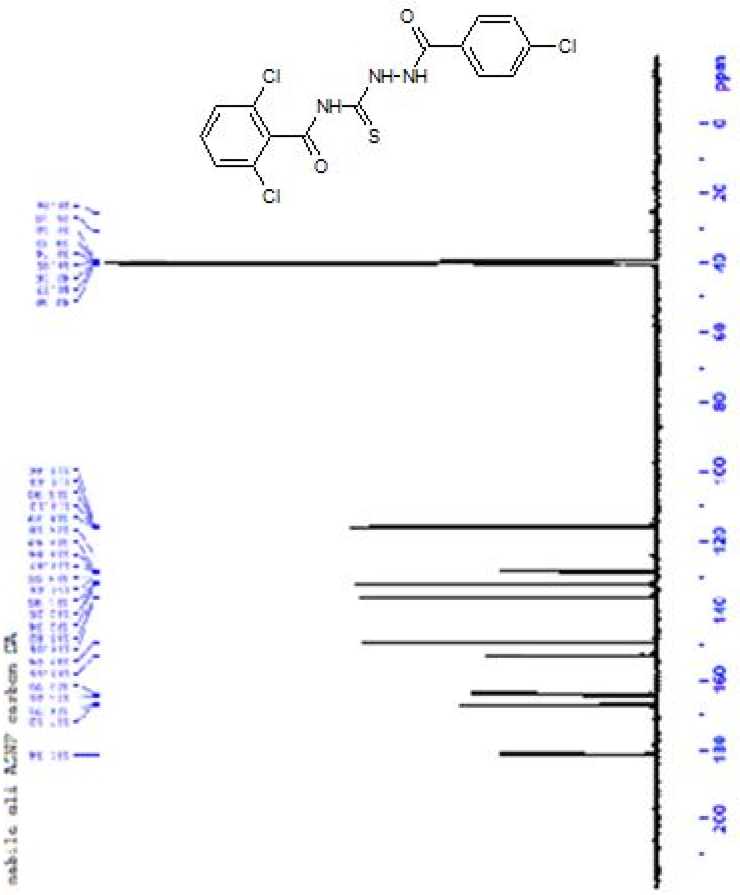

Figure (S11): ^{13}CNMR spectrum of compound b2

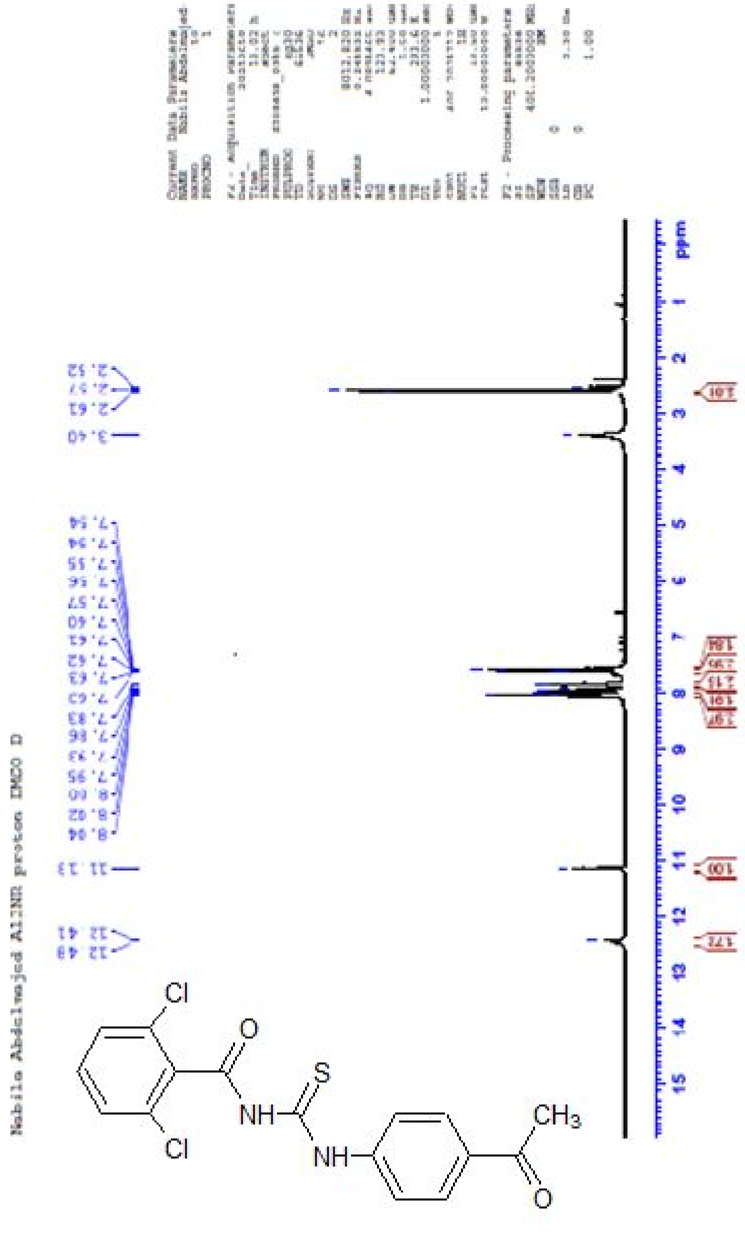

Figure (S12): ^{1}HNMR Spectrum of compound b3

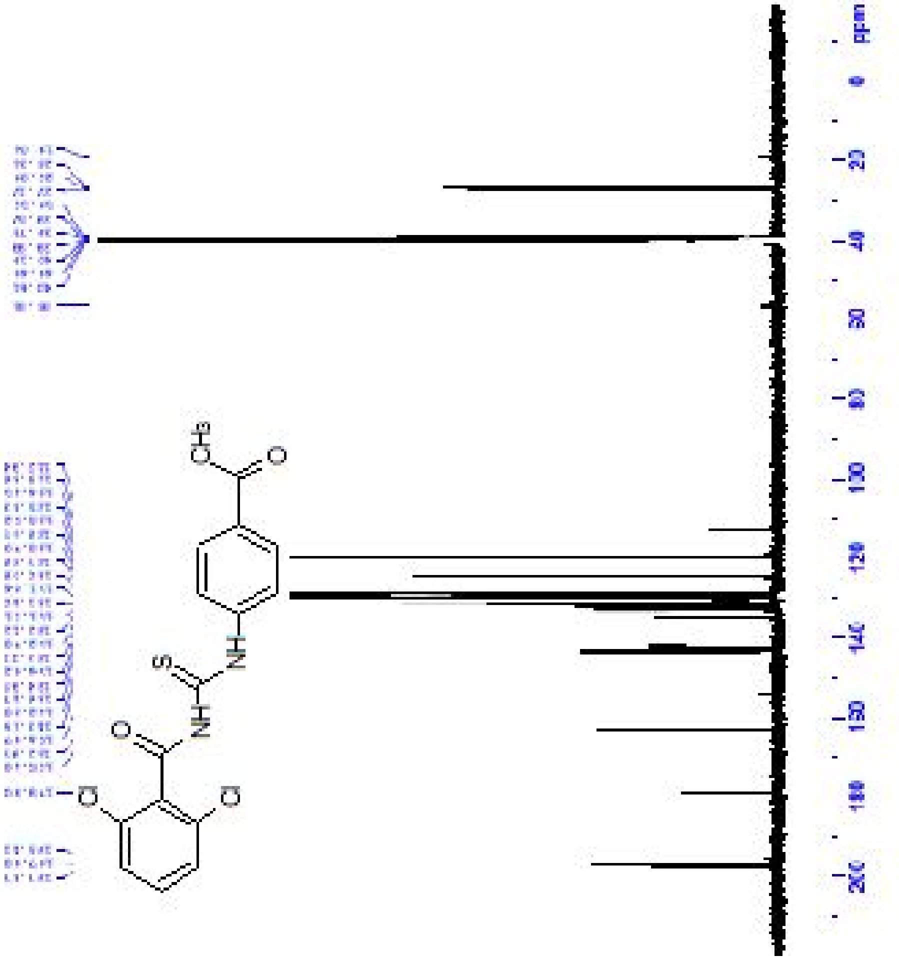

Figure (S13): ^{13}CNMR spectrum of compound b3

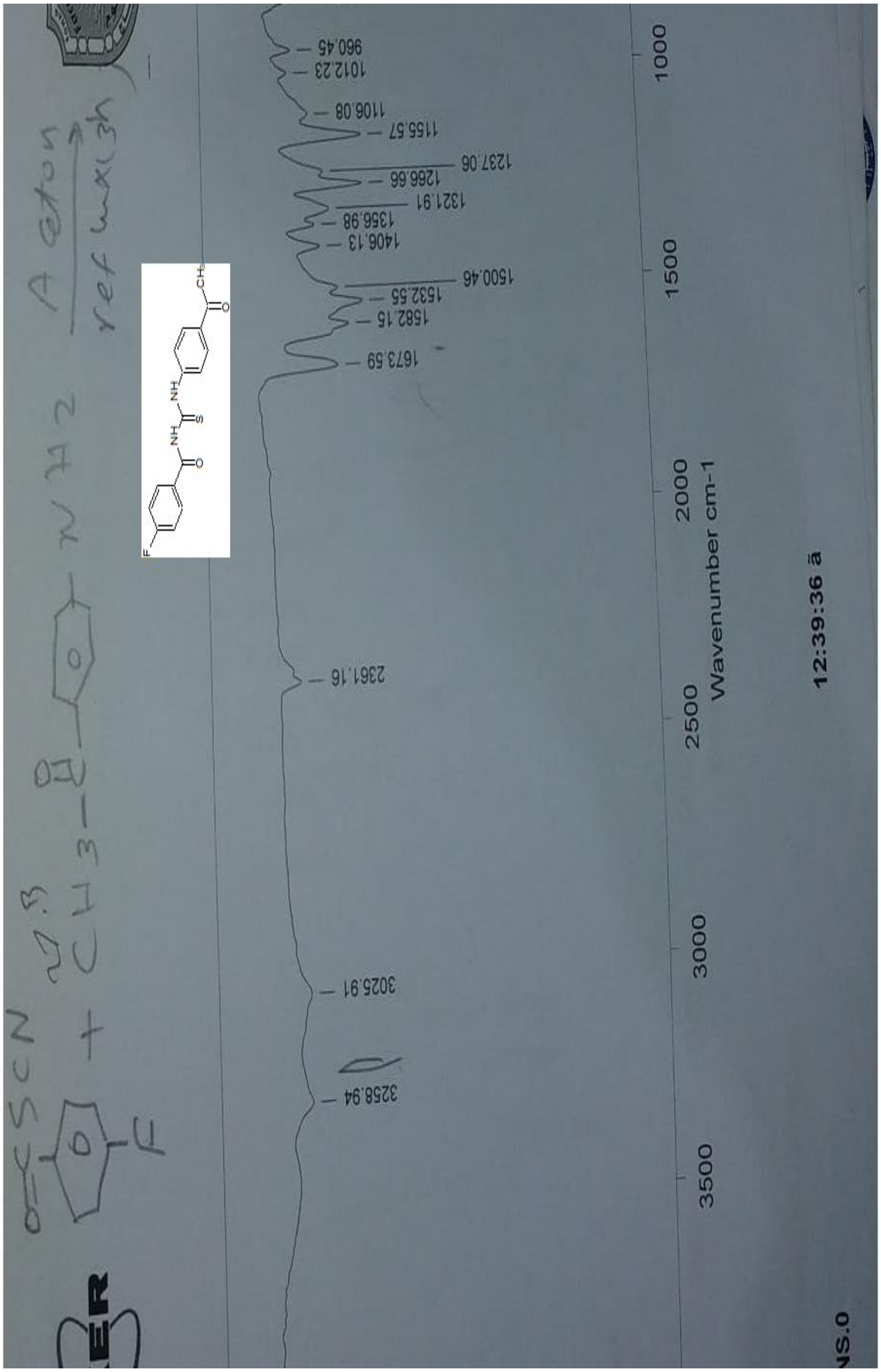

Figure (S14): IR spectrum of compound b4

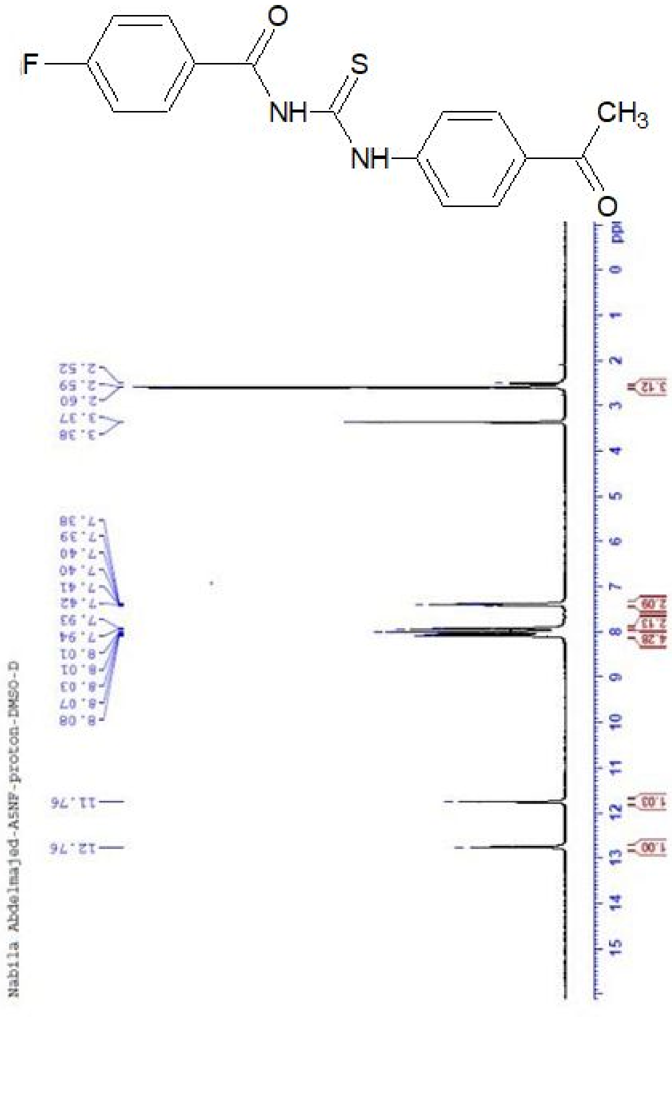

Figure (S15): [1] HNMR spectrum of compound b4

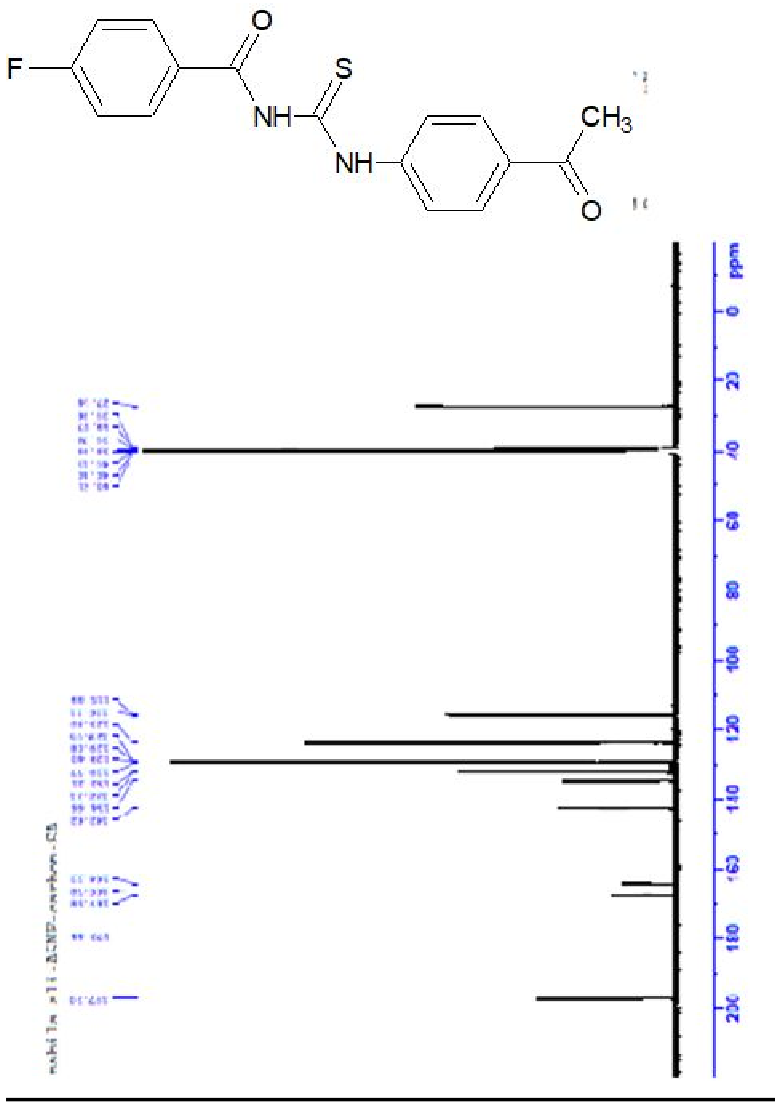

Figure (S16): [13] CNMR spectrum of compound b4

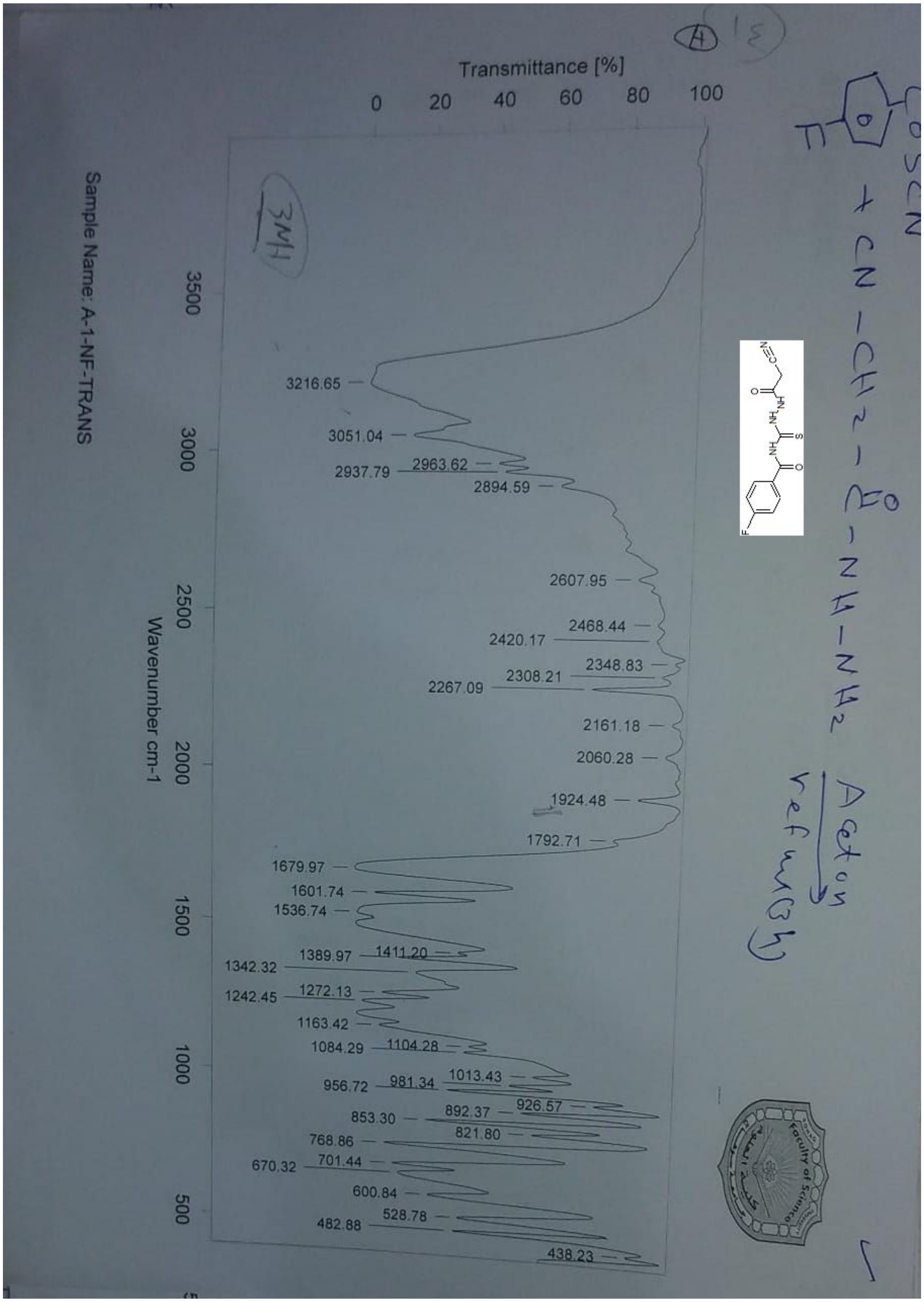

Figure (S17): IR spectrum of compound b5

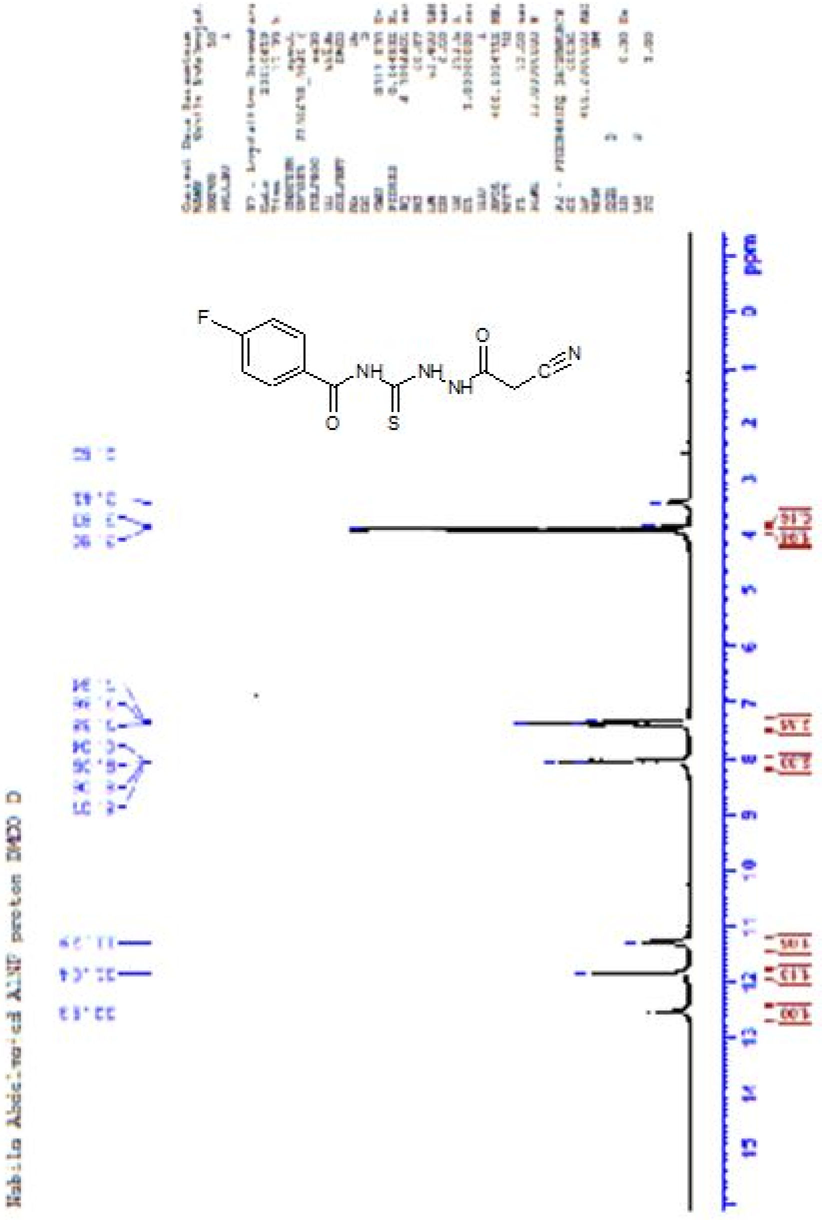

Figure (S18): ^{1}HNMR spectrum of compound b5

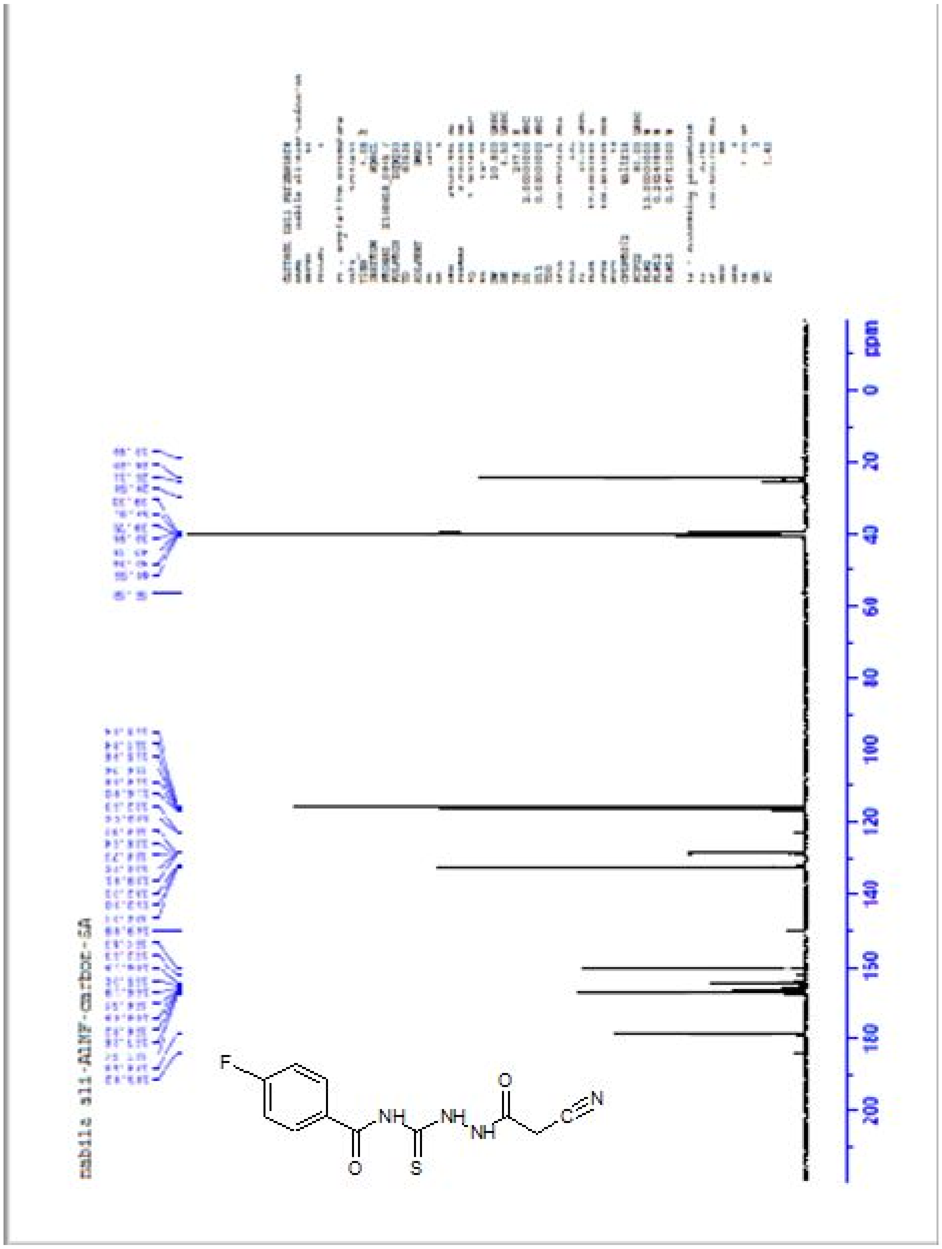

Figure (S19): ^{13}CNMR spectrum of compound b5

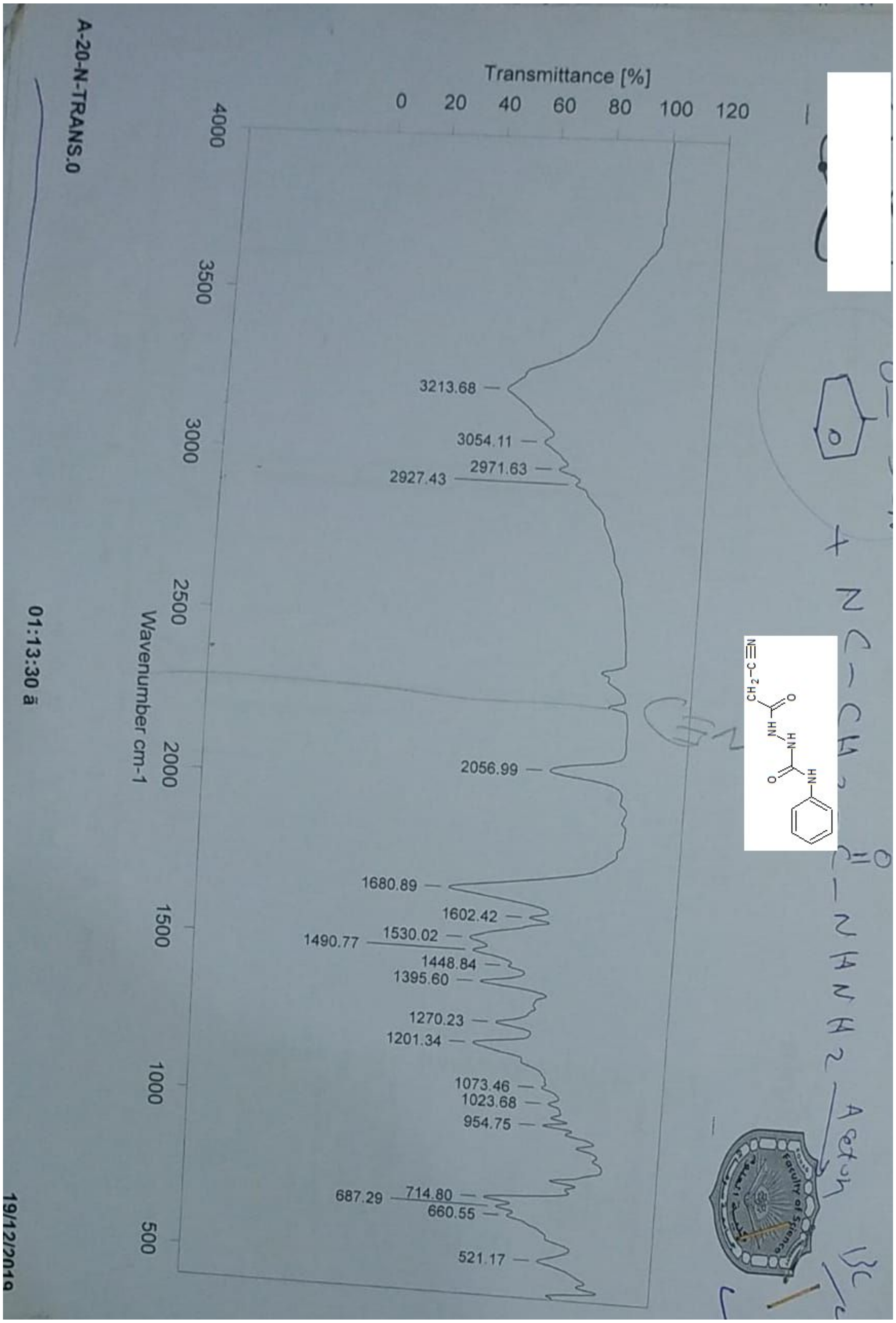

Figure (S20): IR spectrum for compound c2

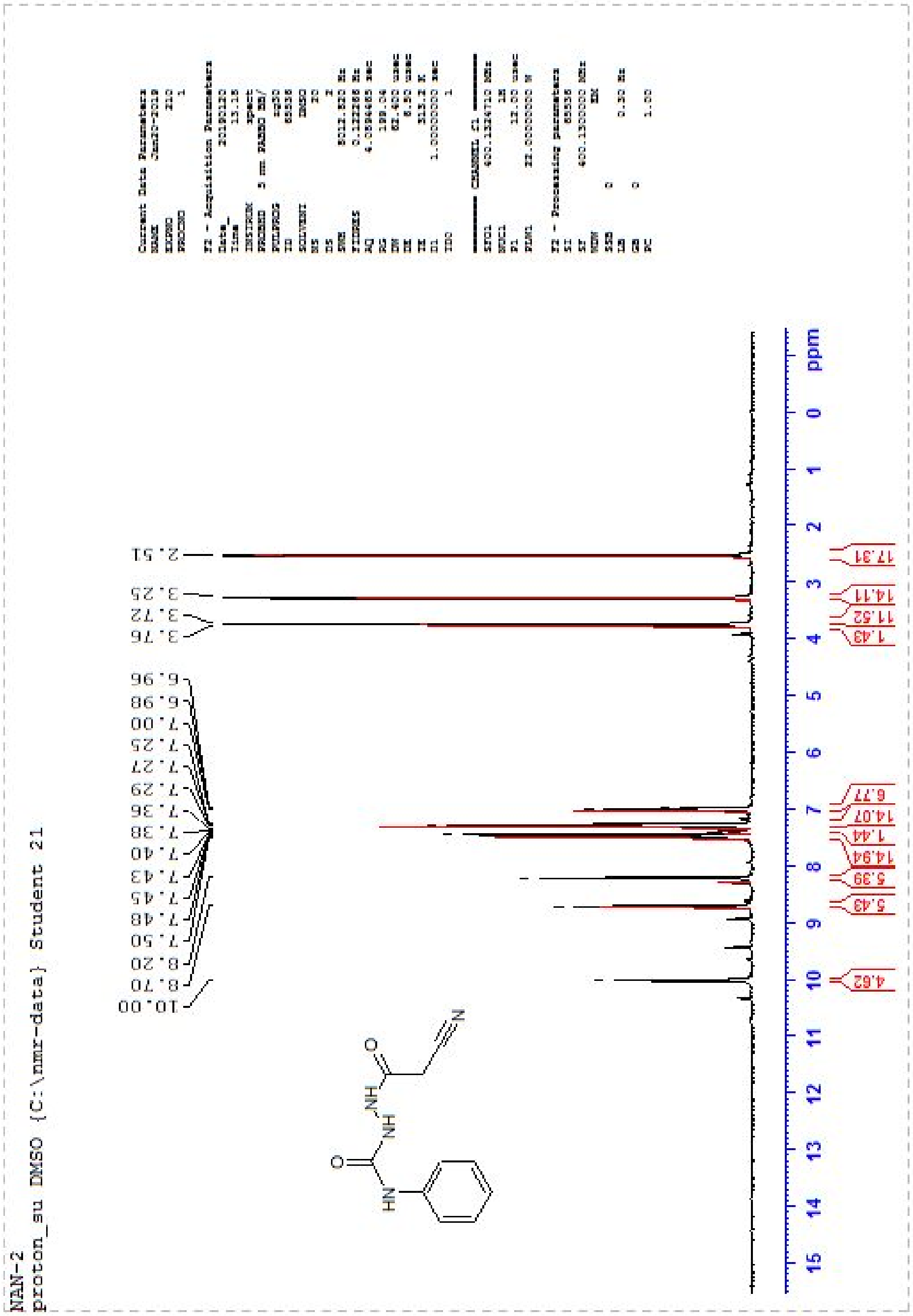

Figure (S21): ^{1}HNMR spectrum for compound c2

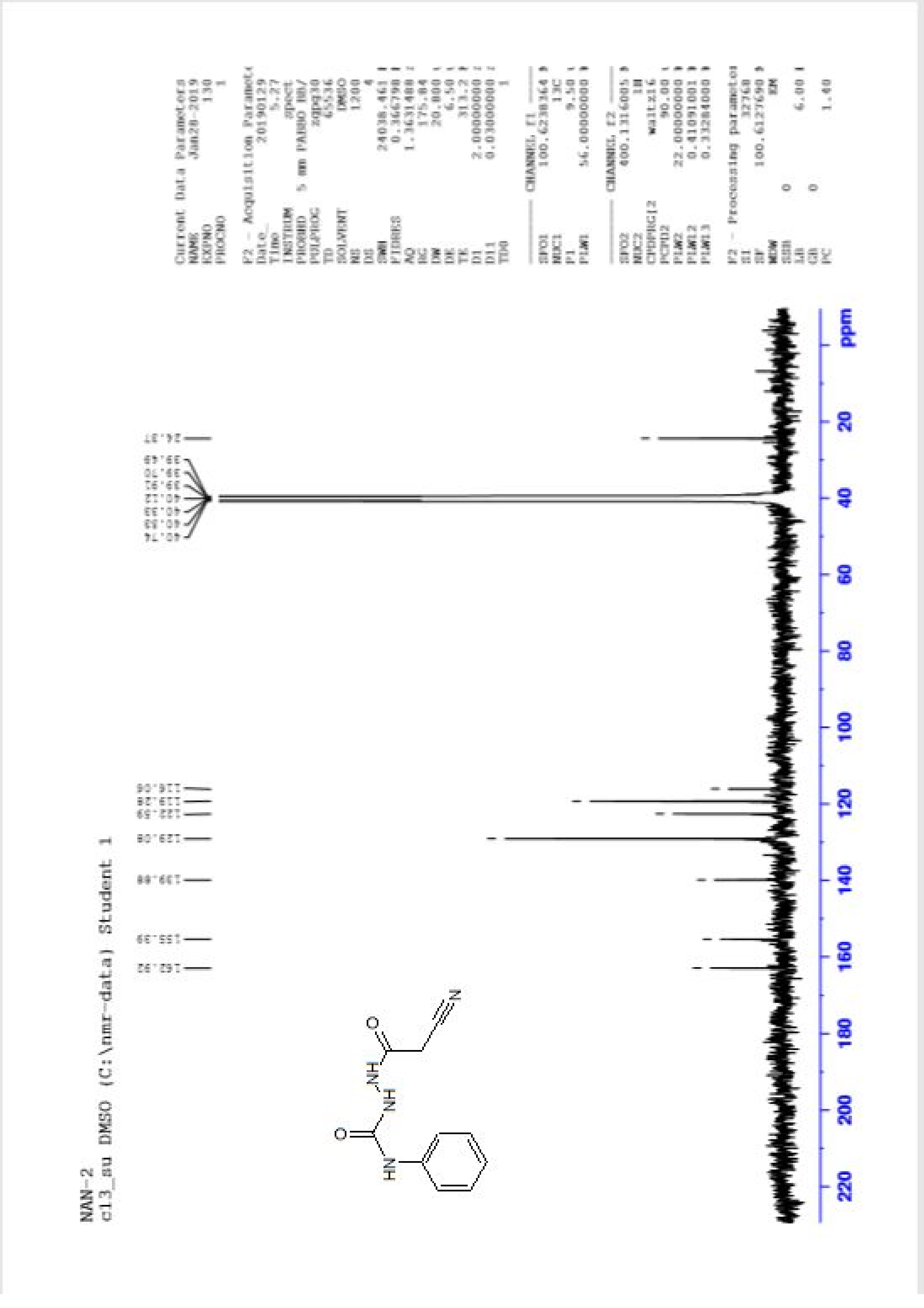

Figure (S22): ^{13}CNMR spectrum for compound **c2**

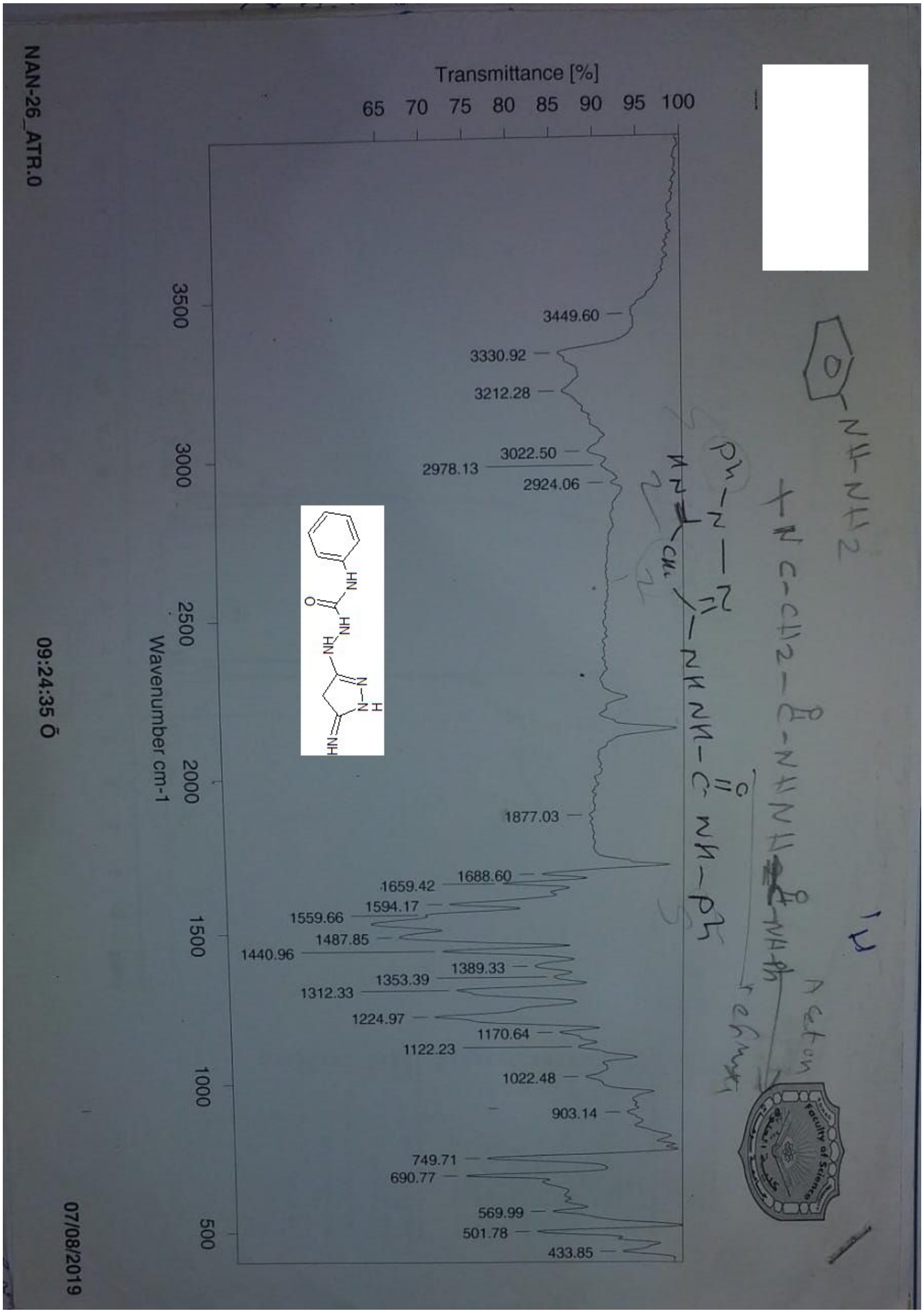

Figure (S23): IR spectrum for compound c3

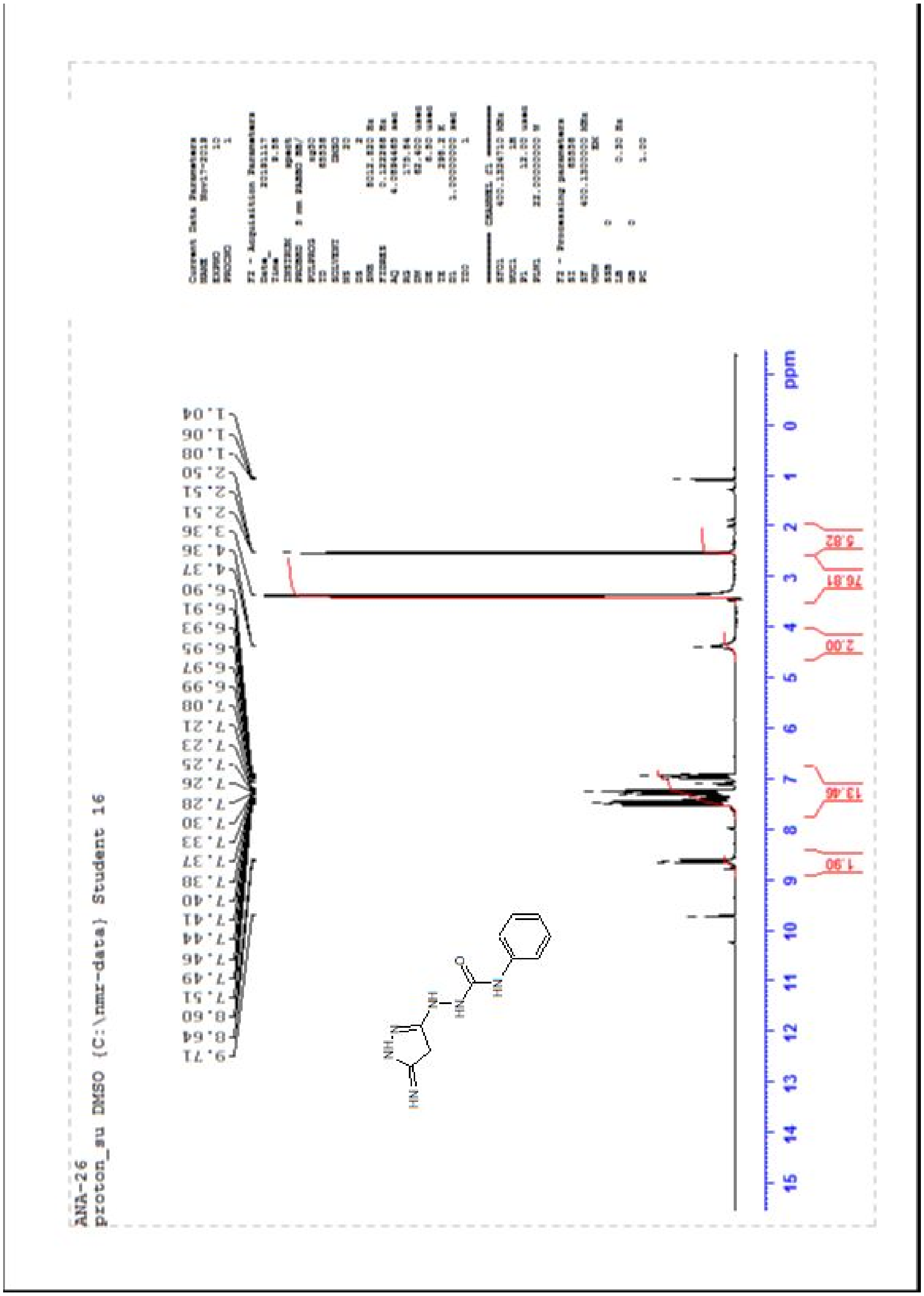

Figure (S24): ^{1}HNMR spectrum for compound c3

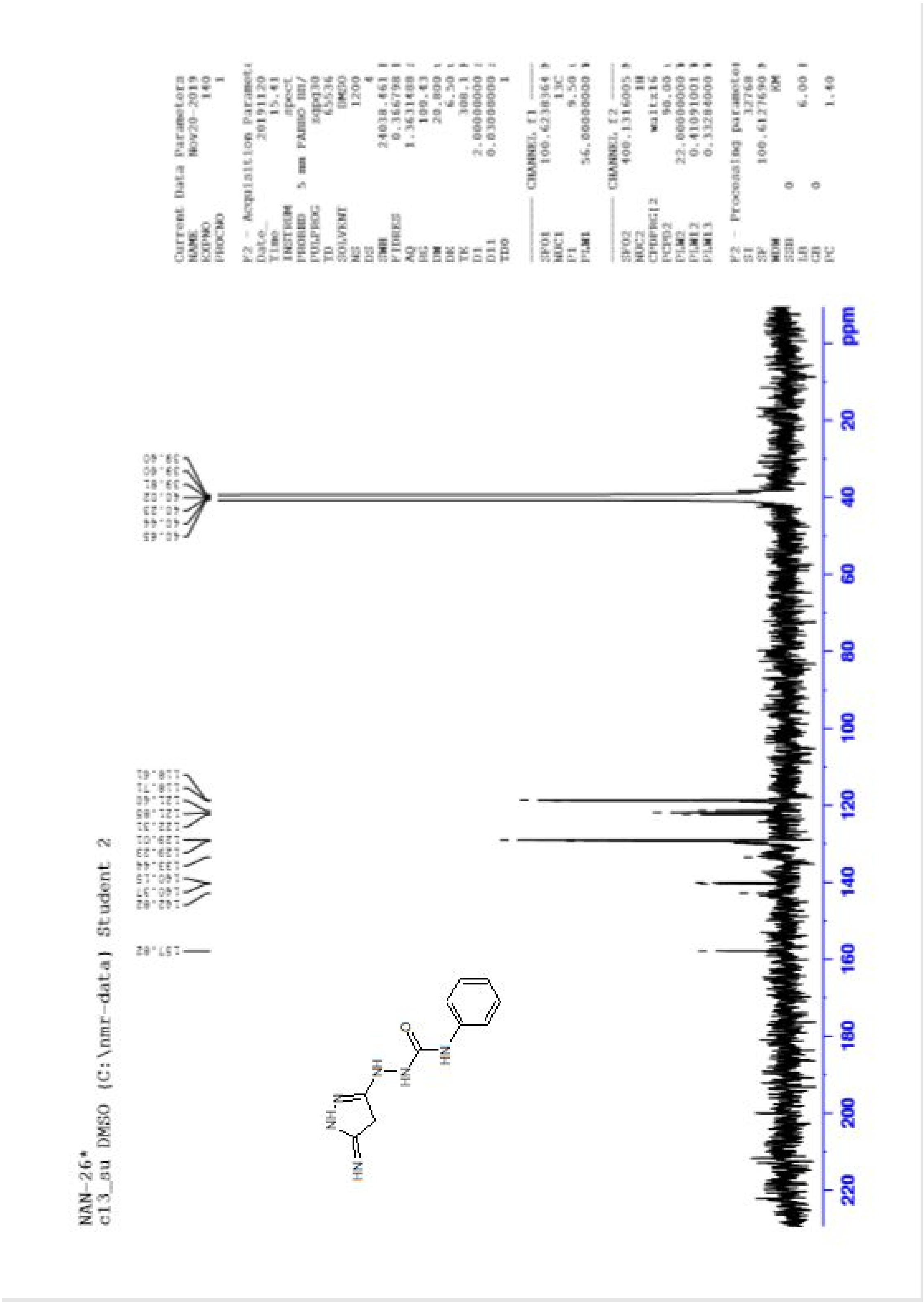

Figure (S25): ^{13}CNMR spectrum for compound c3

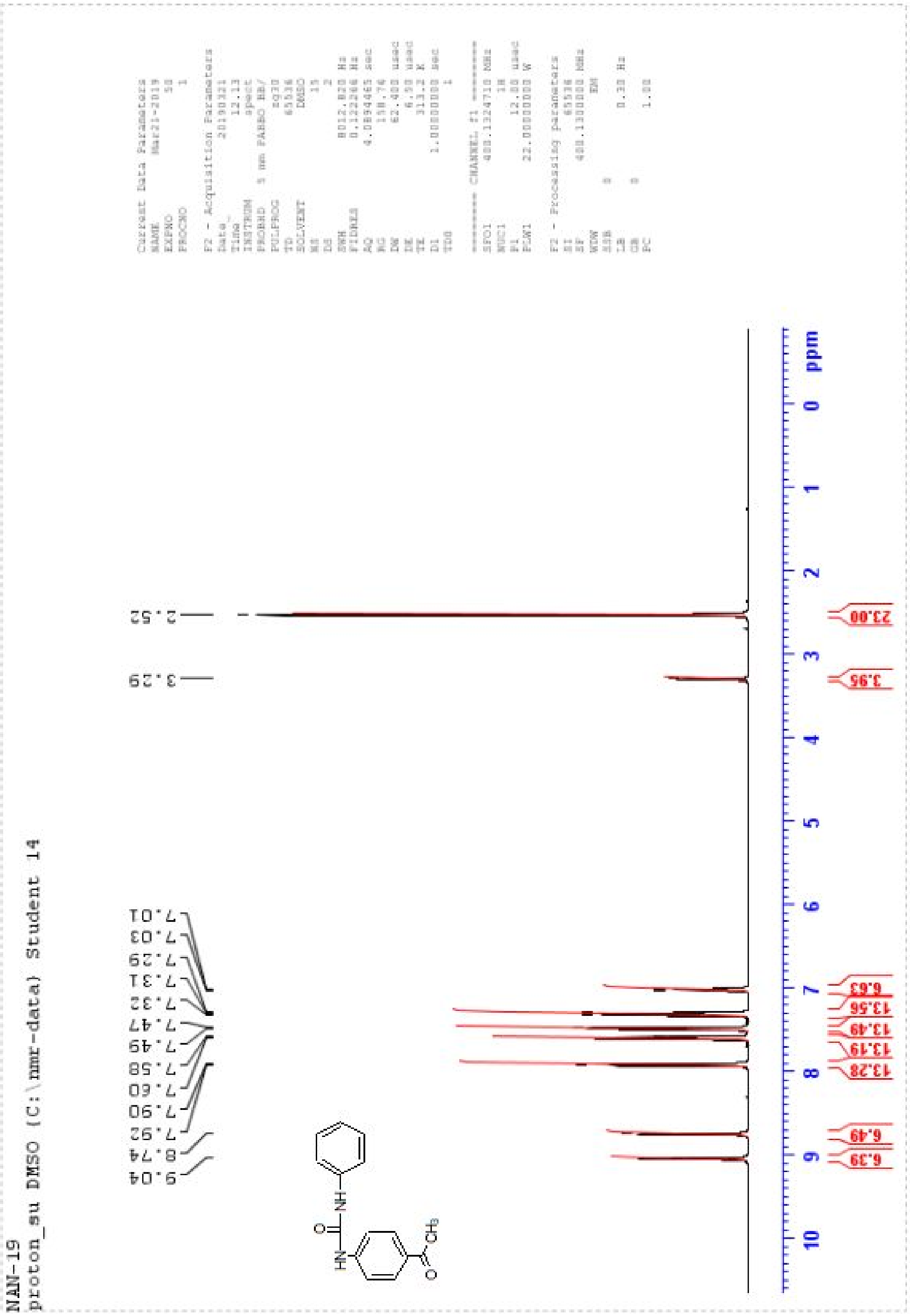

Figure (S26): ^{1}HNMR spectrum for compound c5

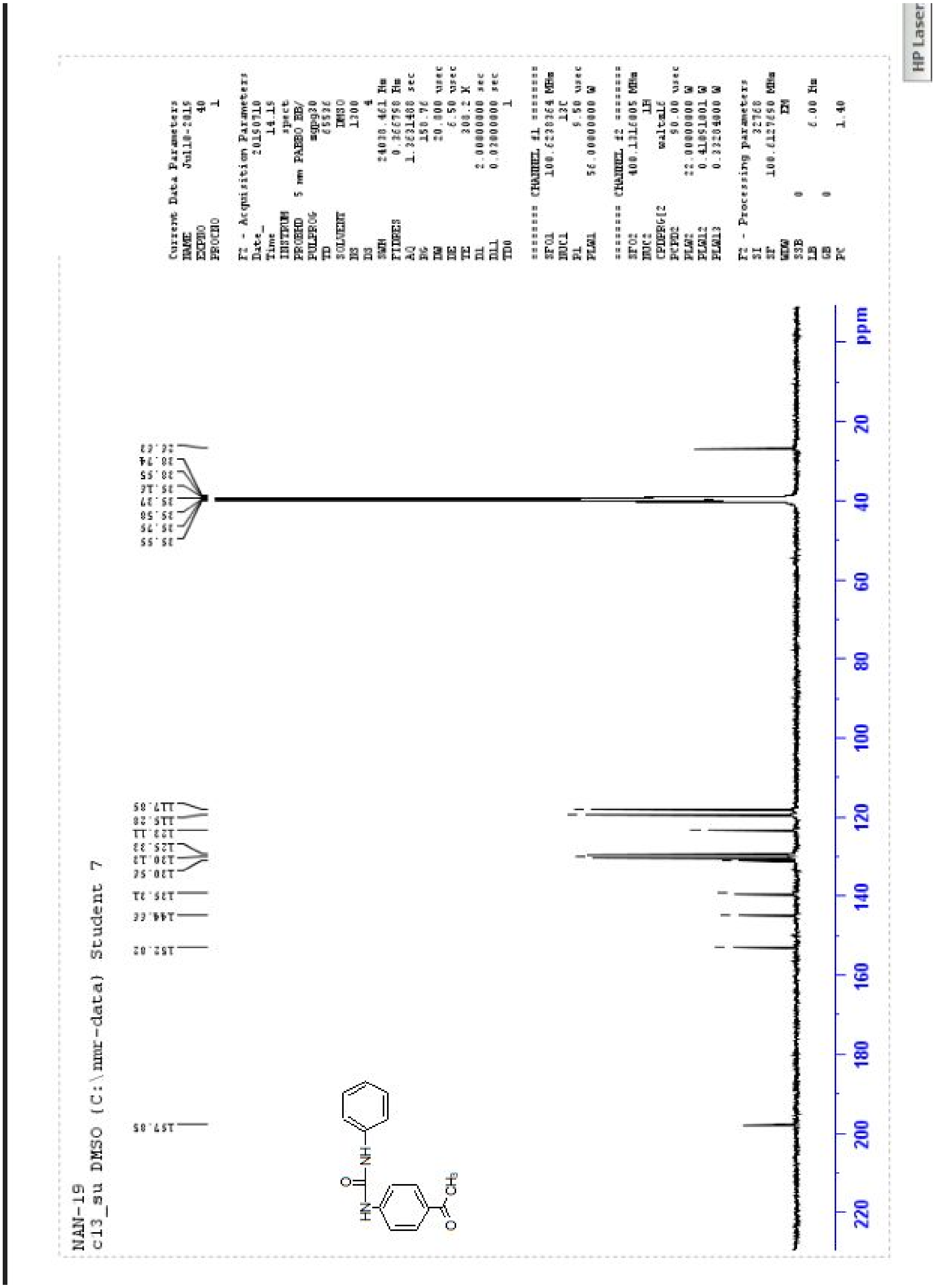

Figure (S27): ^{13}CNMR spectrum for compound c5

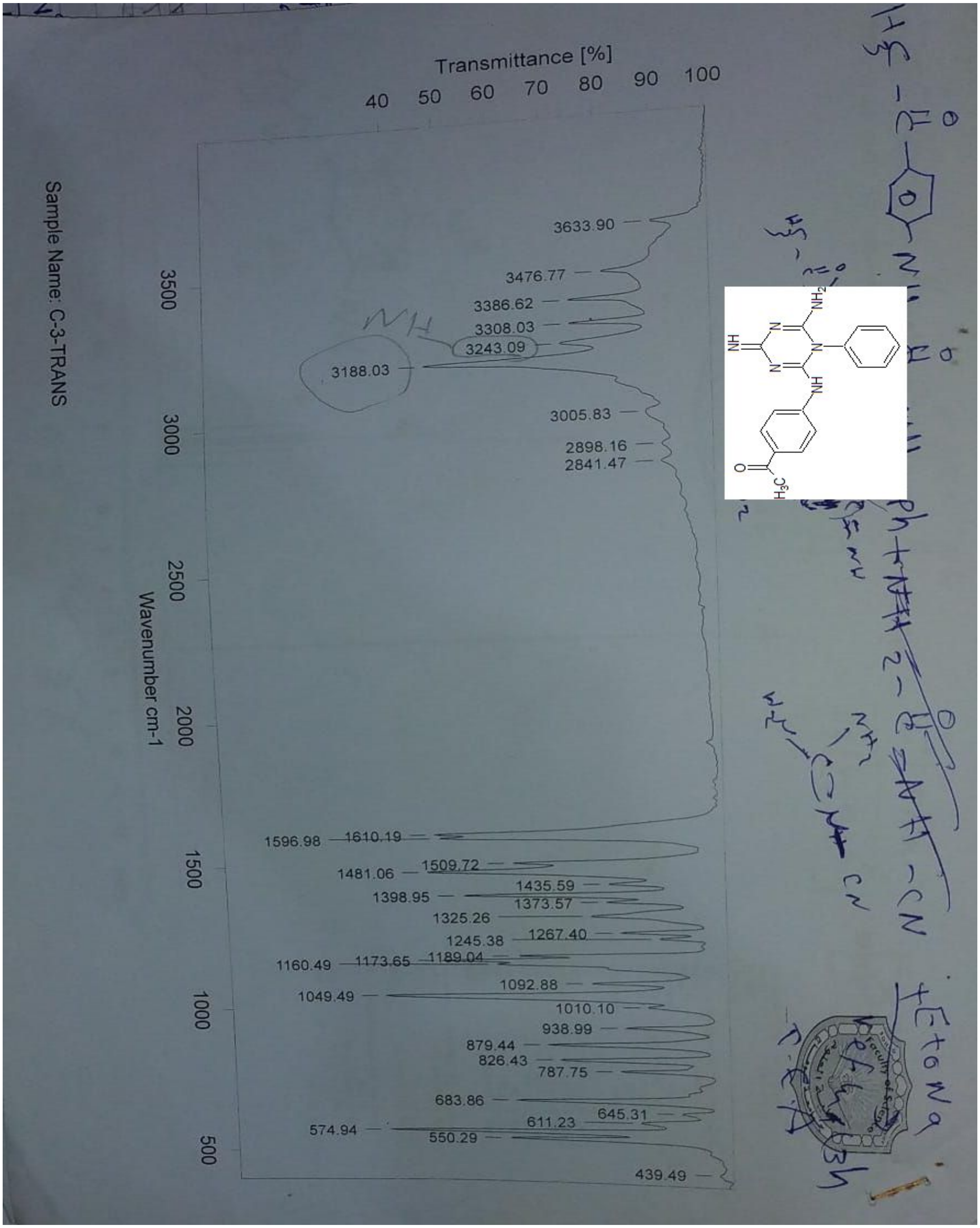

Figure (S28): IR spectrum for compound c6

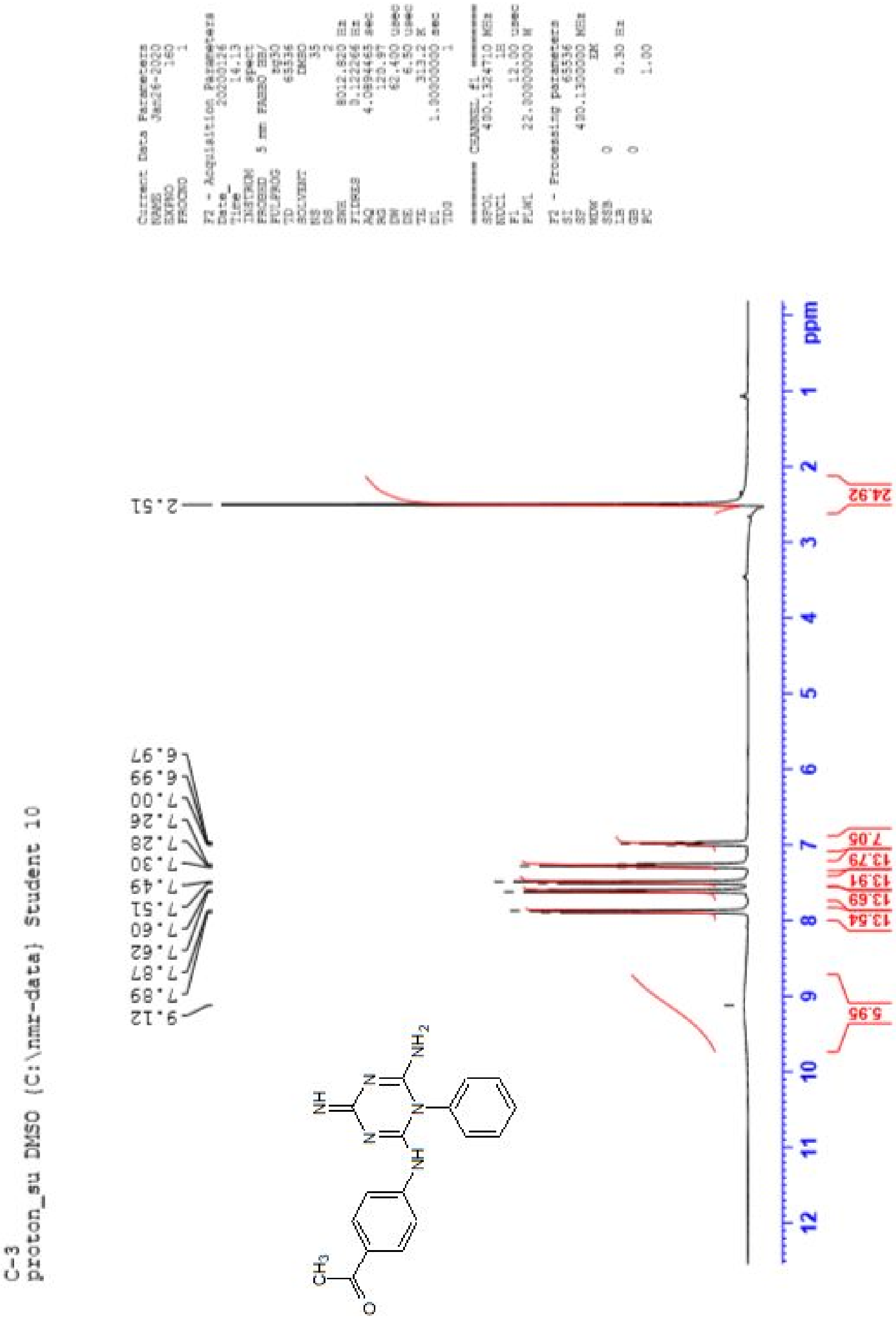

Figure (S29): ^{1}HNMR spectrum for compound c6

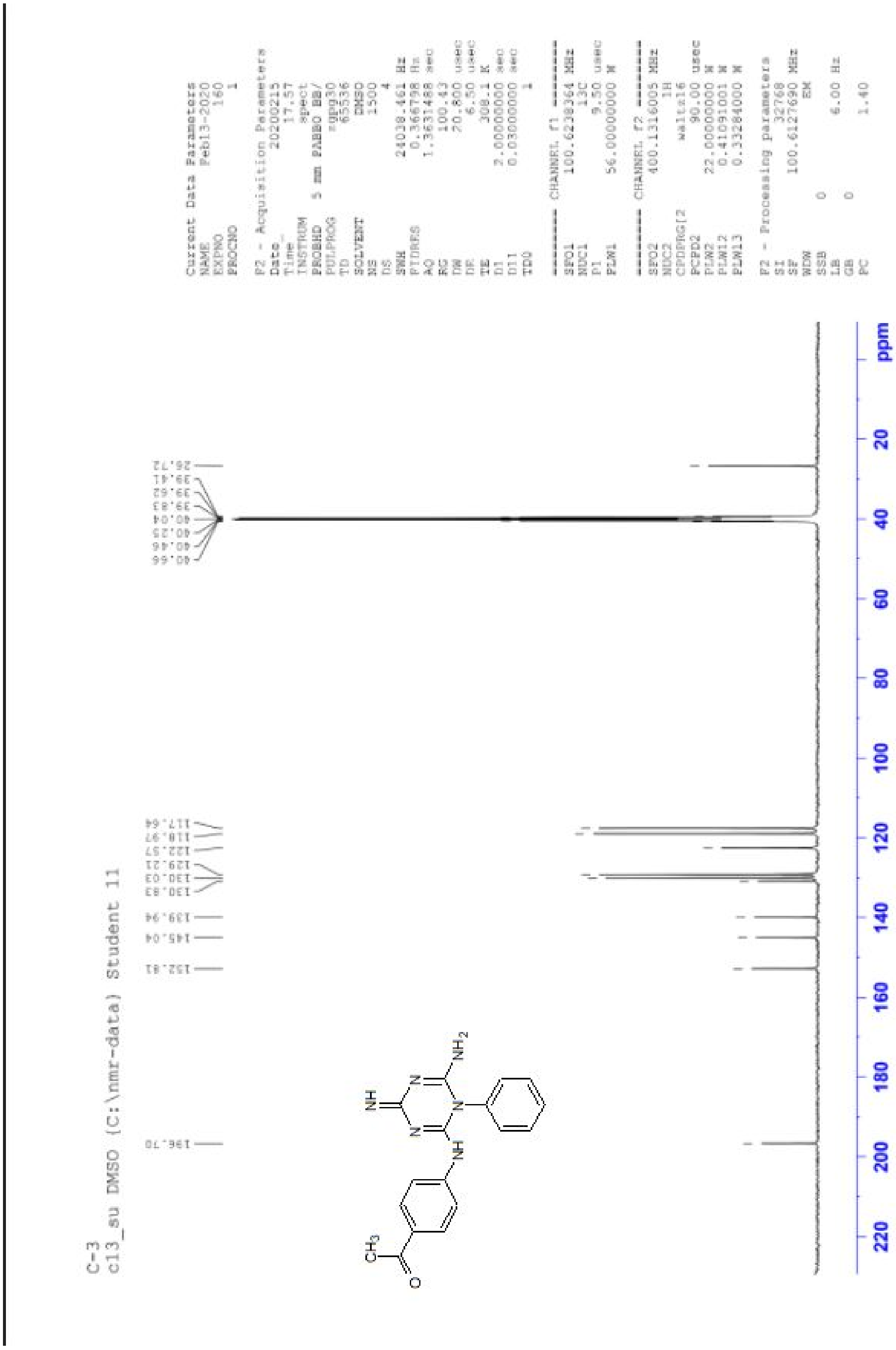

Figure (S30): ^{13}CNMR spectrum for compound c6

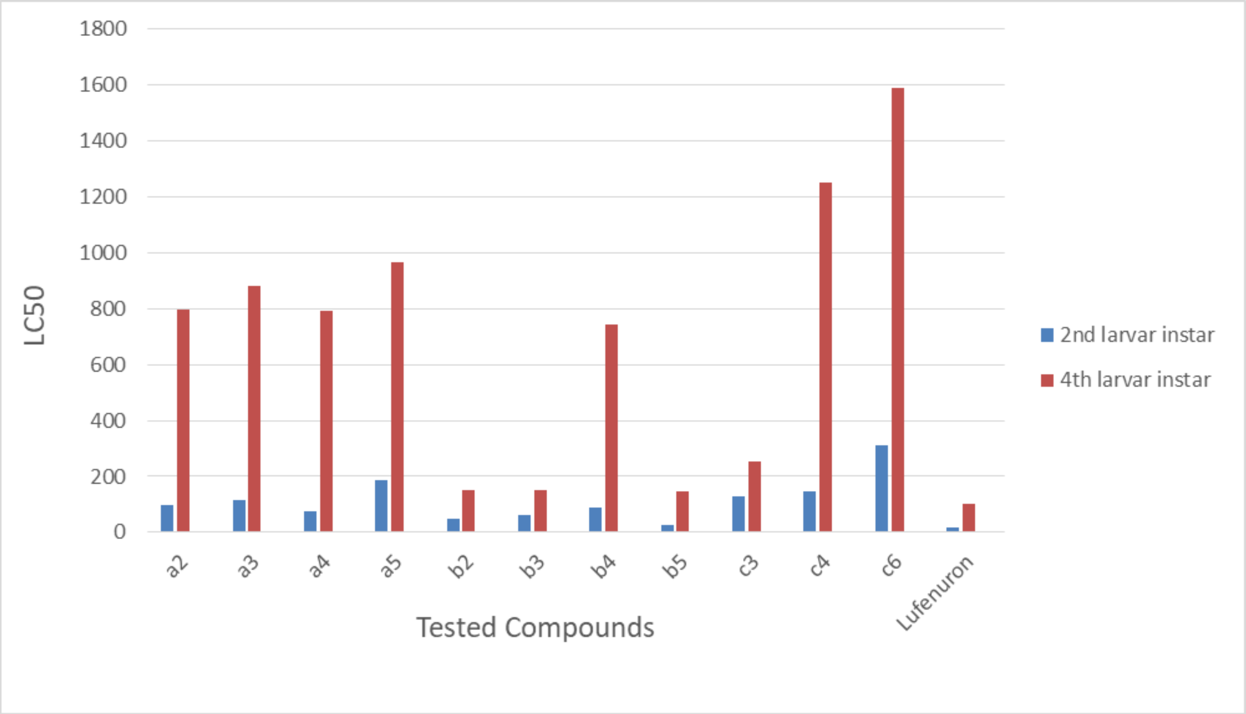

Figure (S31): Insecticidal activity of compounds a2-5, b2-5, c3, c4 and c6 against the 2nd and 4th larvae instar of *S. littoralis* after 72 hours of treatment compared to Lufenuron as the standard insecticide.

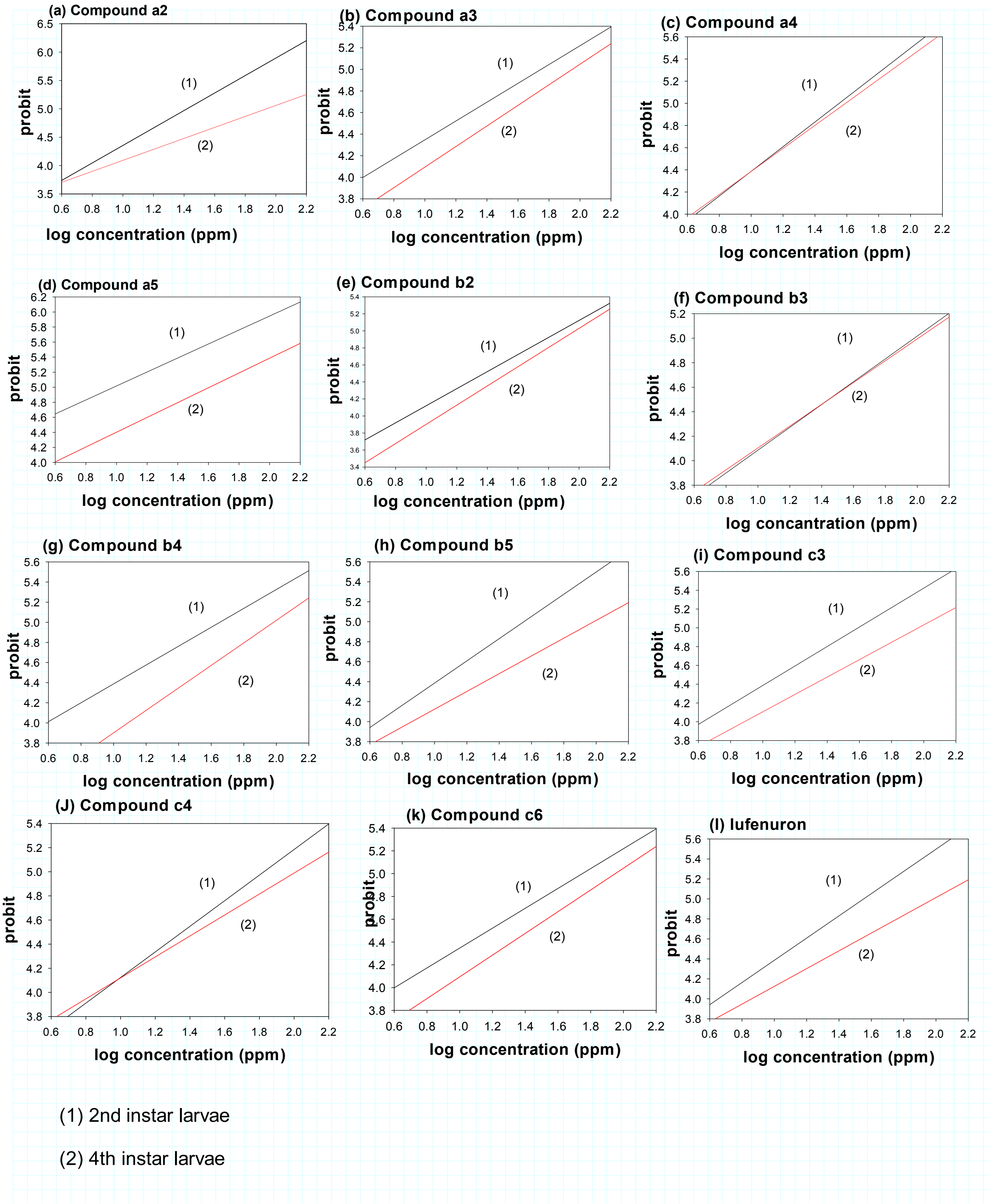

Figure (S32): Insecticidal activities of selective compounds a2-5, b2-5, c3, c4, c6 and Lufenuron as reference insecticide for the 2nd and 4th larvae instar of *S. littoralis* after treatment.

List of Tables

(Table S1): Insecticidal activity of compounds **a2-5, b2-5, c3, c4, c6** and Lufenuron as reference insecticide against for the 2nd and 4th larvae instar of *S. littoralis* after 72 hours of treatment.

2nd instar larvae				4th instar larvae		
Comp.	LC_{50} (ppm)	Slope	Toxic ratio[a]	LC_{50} (ppm)	slope	Toxic ratio
Lufenuron	17.01	0.246±0.0791	1	103.12	0.234 ± 0.083	1
a2	97.37	0.229±0.0813	0.174	798.35	0.418±0.953	0.129
a3	114.10	0.307±0.0993	0.147	881.36	0.853±0.287	0.117
a4	73.35	0.460±0.0805	0.231	793.35	0.418±0.953	0.129
a5	186.48	1.365±0.388	0.279	965.18	2.083±1.0779	0.106
b2	46.35	1.295±0.3923	0.366	148.56	0.302±0.0953	0.694
b3	60.84	0.225±0.8201	0.279	152.06	0.239±0.0985	0.677
b4	86.93	0.231±0.0823	0.196	745.39	3.176±1.184	0.137
b5	26.63	0.246±0.0805	0.638	145.90	0.307±0.0993	0.706
C3	128.41	0.2971±0.0978	0.133	254.41	0.225±0.0820	0.405
C4	145.56	0.307±0.0993	0.116	1253.1	0.422±0.938	0.082
C6	313.11	1.815±1.0141	0.0543	1588.3	0.4.36±1180	0.064
